# Habitual exercise plus dietary supplementation with milk fat globule membrane improves muscle function deficits via neuromuscular development in senescence-accelerated mice

**DOI:** 10.1186/2193-1801-3-339

**Published:** 2014-07-04

**Authors:** Satoshi Haramizu, Takuya Mori, Michiko Yano, Noriyasu Ota, Kohjiro Hashizume, Atsuko Otsuka, Tadashi Hase, Akira Shimotoyodome

**Affiliations:** Biological Science Laboratories, Kao Corporation, Tochigi, Japan

**Keywords:** Aging, Milk fat globule membrane, Muscle function, Neuromuscular junction

## Abstract

We examined the effects of habitual exercise plus nutritional intervention through consumption of milk fat globule membrane (MFGM), a milk component, on aging-related deficits in muscle mass and function in senescence-accelerated P1 mice. Combining wheel-running and MFGM (MFGMEx) intake significantly attenuated age-related declines in quadriceps muscle mass (control: 318 ± 6 mg; MFGMEx: 356 ± 9 mg; *P* < 0.05) and in contractile force (1.4-fold and 1.5-fold higher in the soleus and extensor digitorum longus muscles, respectively). Microarray analysis of genes in the quadriceps muscle revealed that MFGMEx stimulated neuromuscular development; this was supported by significantly increased docking protein-7 (Dok-7) and myogenin mRNA expression. Treatment of differentiating myoblasts with MFGM-derived phospholipid or sphingolipid fractions plus mechanical stretching also significantly increased Dok-7 mRNA expression. These findings suggest that habitual exercise plus dietary MFGM improves muscle function deficits through neuromuscular development, and that phospholipid and sphingolipid in MFGM contribute to its physiological actions.

**Electronic supplementary material:**

The online version of this article (doi:10.1186/2193-1801-3-339) contains supplementary material, which is available to authorized users.

## Background

Aging skeletal muscle is characterized by progressive loss of muscle mass and function (Breen and Phillips 2011). This age-related deficit, known as sarcopenia, has a profound effect on quality of life in the elderly and increases the risk of morbidity, disability, and death (Janssen et al. 2004). Despite the high prevalence and clinical relevance of sarcopenia, the exact biochemical and molecular mechanisms of muscle wasting during aging are not fully understood. The etiology of sarcopenia is multi-factorial and involves both intrinsic and extrinsic factors (Sinha-Hikim et al. 2013; Tan et al. 2012).

Accumulated evidence from both animal and human studies suggests that skeletal muscle aging is strongly associated with degeneration of motor neurons, followed by changes in the structural and functional integrity of the neuromuscular junction (NMJ), along with functional denervation, and loss of motor units (Chai et al. 2011; Manini and Clark 2012; Valdez et al. 2010). Aging muscle fibers undergo denervation and reinnervation cycles that lead to remodeling of the motor units (Larsson 1995). Preferential denervation of the fast-twitch fibers and reinnervation by axonal sprouting from slow motor neurons result in the conversion of type II fast fibers to type I slow fibers (Balice-Gordon 1997; Kadhiresan et al. 1996). When denervation outpaces reinnervation, some of the muscle fibers degenerate and atrophy occurs in the remaining fibers (Rowan et al. 2012). Functional loss of NMJs and muscle mass ultimately contributes to compromised contractile function.

Milk is the main source of nutrition in newborn mammals. Recent findings in young adults have demonstrated that consumption of whole milk after resistance training can promote muscle protein synthesis and inhibit protein breakdown, leading to improved net muscle protein balance (Elliot et al. 2006; Josse et al. 2010; Wilkinson et al. 2007). In addition, milk protein has beneficial effects including suppressing postprandial glycemia and promoting changes in body composition for metabolic health (McGregor and Poppitt 2013). Milk contains approximately 3% to 5% fat, which is distributed in the form of tiny, spherical droplets or globules stabilized in the form of an emulsion. The diameter of the fat globule ranges from 0.2 to 15 μm, with an average of about 4 μm. The triglyceride core of the fat globules in milk is surrounded by a thin membrane called the milk fat globule membrane (MFGM). This membrane, which is about 10 to 20 nm in cross-section, acts as an emulsifier and protects the globules from coalescence and enzymatic degradation. The MFGM contains unique polar lipids and membrane-specific proteins (Cavaletto et al. 2008). The polar lipids in milk, which consist of phospholipids and sphingolipids, are located mainly (60% to 70%) in the MFGM. Sphingolipids (highly bioactive molecules present mainly in polar lipids of animal origin) account for up to one third of the MFGM polar lipid fraction. Oshida et al. have shown that dietary sphingomyelin contributes to myelination in the central nervous system of developing rats (Oshida et al. 2003). Loss of myelinated nerve fibers, along with several abnormalities involving myelinated fibers, such as demyelination and myelin balloon figures, has been observed in elderly subjects. Deterioration of the myelin sheaths during aging may affect the functional properties of the peripheral nervous system; it may cause a decline in the conduction velocity of motor neurons and thereby in muscle strength and mass. Even though scientific evidence on the nutritional benefits of MFGM proteins and sphingolipids is accumulating (Vesper et al. 1999), the nutritional aspects and physiological functions of MFGM have not been fully investigated.

The senescence-accelerated mouse (SAM) exhibits several accelerated aging characteristics and is widely used in research on aging (Takeda et al. 1981). The SAM consists of two types of strain, namely the senescence-accelerated prone mouse (SAMP) and the senescence-accelerated resistant mouse (SAMR). Compared with SAMR strains, which are used as controls that age normally, SAMP strains show rapid progression of senescence, higher oxidative stress, decreased behavioral activity, and a shorter median life span (Takeda et al. 1991, 1997; Takeda 1999; Hosokawa 2002). These characteristics observed in SAMP strains are similar to those observed in humans with normal aging; therefore, SAMP strains are useful models of human aging. SAMP strains might also be useful for studying muscle aging. Sakakima et al. (2004) demonstrated that sarcopenia and age-associated morphologic changes in the leg muscles occur earlier in SAMP1 mice than in normal ICR mice. Derave et al. (2005) reported reduced muscle mass, selective type-II fiber atrophy, and reduced contraction speed in the soleus muscle of SAMP8 mice. There is, thus, support for using this model to study skeletal muscle aging.

Our previous studies have shown that dietary green tea extract (GTE) plus habitual exercise improves skeletal muscle function and metabolism in mice, including in SAMP1 mice (Murase et al. 2005, 2006a, [b], 2008; Shimotoyodome et al. 2005). These findings led us to investigate whether, and how, habitual exercise plus nutritional intervention improves skeletal muscular physiology.

Here, we investigated whether long-term consumption of MFGM proteins and lipids combined with habitual exercise could prevent age-related deficits in muscle mass and function. In light of our finding that a combination of dietary supplementation with MFGM and voluntary wheel-running prevented age-related declines in skeletal muscle mass and strength in SAMP1, we also attempted to elucidate the mechanisms underlying these improvements after long-term MFGM consumption.

## Methods

### Materials

MFGM was purchased from MEGGLE Japan Co. Ltd (Tokyo, Japan). The composition of the MFGM was 43.8% protein, 37.1% fat, 10.3% carbohydrate, 13.6% lactose, 16.6% phospholipids (4.71% phosphatidylcholine, 5.2% phosphatidylethanolamine, 1.32% phosphatidylinositol, 1.74% phosphatidylserine, 3.0% sphingomyelin, and others), 4.3% ash, 2.4% minerals, 4.5% moisture, and 1.9% others. The composition of the fatty acids was 31.6% C18:1, 23.8% C16:0, 12.9% C18:0, 7.5% C14:0, 4.3% C18:2(n-6), 2.1% C12:0, 1.6% C4:0, 1.6% C10:0, 1.5% C16:1, 1.2% C6:0, and <1.0% others. The content (all in g/100 g) of glutamic acid was 8.44, of leucine 4.46, aspartic acid 3.82, lysine 3.69, proline 3.49, serine 2.83, valine 2.54, threonine 2.33, isoleucine 2.26, phenylalanine 2.1, tyrosine 1.86, arginine1.85, alanine 1.66, histidine 1.35, methionine 1.16, glycine 1.04, tryptophan 0.71, and cysteine 0.57. Milk-derived sphingomyelin was purchased from NOF Corporation (Tokyo, Japan). Phospholipid and sphingolipid fractions were also prepared from MFGM. In brief, the MFGM was homogenized in an ice-cold chloroform–methanol mixture (2:1) by using a homogenizer (TK autohomomixer; Tokusyukika Kogyo Co. Ltd., Osaka, Japan). The soluble fraction obtained was subjected to acetone precipitation to separate the polar lipids. The phospholipid fraction (PLF) was then purified by using column chromatography over silica gel (Yamazen Hi-Flash silica gel column; Yamazen Corp., Osaka, Japan). Phosphatidylcholine, phosphatidylethanolamine, phosphatidylserine, glucosylceramide, and lactosylceramide in the purified PLF were detected by using thin-layer chromatography (TLC) analysis (chloroform–methanol–water; 65:16:2). After alkaline hydrolysis, the sphingolipid fraction (SLF) was obtained as an acetone-insoluble fraction after acetone precipitation. Glucosylceramide, lactosylceramide, and sphingomyelin were detected by TLC analysis of the SLF. Quantitative analysis by using high-performance liquid chromatography revealed that the SLF was 52% sphingomyelin. GTE was prepared and analyzed as described previously (Haramizu et al. 2011a, 2011b). The total polyphenol (catechin) content of the GTE was 81%, and the caffeine content was 0.1%. The polyphenols were made up of epigallocatechin gallate (41%), epigallocatechin (23%), epicatechin gallate (12%), epicatechin (9%), gallocatechin (7%), gallocatechin gallate (4%) and others (4%).

### Animals and experimental design

SAMP1 exhibit several characteristics of accelerated aging and are widely used in aging research (Sakakima et al. 2004). ICR mice are widely used as a control strain in experiments on SAMP1 (Lee et al. 2013; Nagano et al. 2000). Male 15-week-old SAMP1 and ICR mice were purchased from Japan SLC, Inc. (Hamamatsu, Japan) and maintained under controlled conditions of temperature (23 ± 2°C), humidity (55% ± 10%), and lighting (0700 to 1900 h). The mice were fed a laboratory chow (CE-2, CLEA Japan, Inc., Tokyo, Japan) and had free access to drinking water to acclimate to the housing conditions for 2 months. At the age of 23 wk, all mice were weighed and those that were self-injurious (i.e., those with visible injury) were removed from the study. SAMP1 and ICR mice whose body weights were 20% heavier and 9% lighter, respectively, than the average were also removed to minimize individual differences in body weight. Then, 40 of 80 SAMP1 and 8 of 12 ICR mice with similar body weights were selected. The SAMP1 mice were randomly allocated to five groups, namely the control group, which was fed a control diet containing 5.5% fat (w/w), 6% casein, 75.6% potato starch, 8.4% cellulose, 3.5% minerals, and 1% vitamins; the MFGM group, which was fed an MFGM diet consisting of the control diet supplemented with 1% MFGM; the GTE group, which was fed a GTE diet consisting of the control diet supplemented with 0.5% GTE; the MFGMEx group, which was fed the MFGM diet and also given habitual exercise; and the GTEEx group, which was fed the GTE diet and also given habitual exercise (n = 8 per group).

ICR mice, which had previously been used as a control strain for comparison with SAMP1 were used as normally aging mice. The ICR mice were used as normally aging mice and were fed the control diet (n = 8). For 20 wk (from 23 to 43 wk of age), the mice were allowed *ad libitum* access to water and one of the following powdered diets: the control diet (control and ICR groups) or each experimental diet with or without exercise (MFGM, MFGMEx, GTE, and GTEEx groups). All mice were individually housed in regular plastic cages (TP-106; 175 × 245 × 125 mm, Toyoriko, Tokyo, Japan), each of which had a nest box (Shepherd Specialty Papers, Watertown, TN) to reduce stress. The cages of habitual exercise group had a running wheel (SW-15 mg; MELQUEST, Toyama, Japan), whereas those of the non-habitual-exercise groups did not. Dietary intake was measured throughout the experimental period by subtracting the remaining food weight from the initial weight of the food given on the previous feeding day. All animal experiments were conducted in the Experimental Animal Facility of Kao Corporation R&D Department. The study was approved by the Animal Care Committee of the Kao Tochigi Institute. All experiments strictly followed the guidelines of that committee.

### Cell culture and mechanical stretching by using cyclic strain

Murine C2C12 myoblasts (EC91031101) were obtained from the European Collection of Cell Cultures (Dainippon Sumitomo Pharma Biomedical, Osaka, Japan). The cells were plated onto flexible-bottomed plates (Bioflex Plates Collagen 1, Flexcell International Corp., Hillsborough, NC) coated with 1 mg/mL poly-L-lysine (Sigma-Aldrich Japan, Tokyo, Japan) and fibronectin (1:100, Sigma-Aldrich Japan) and maintained in an atmosphere of 95% air – 5% CO_2_ at 37°C in Dulbecco’s modified eagle medium (DMEM) supplemented with 10% fetal bovine serum and 10 ml/L Antibiotic-Antimycotic mixture (Gibco, Grand Island, NY). For differentiation into myotubes, C2C12 myoblasts were grown to subconfluence on the plates; the culture medium was then replaced with DMEM containing 2% heat-inactivated horse serum (Gibco) supplemented or not supplemented with 0.01% MFGM, 0.001% or 0.005% PLF, SLF, or sphingomyelin. During differentiation, the cells were subjected to cyclic equibiaxial stretching consisting of 10% elongation at 0.5 Hz, with 1 h on and 5 h off for 72 h, by using a Flexcell FX-5000 Tension System (Flexcell International Corp.), as described previously (Zhang et al. 2007). The culture media were replaced with fresh media once a day. At the end of 72 h, the cells were washed with ice-cold phosphate-buffered saline once, homogenized with a QIAshredder (Qiagen K.K., Tokyo, Japan), and subjected to RNA extraction. Total RNA was extracted from frozen samples (n = 6) by using an RNeasy Mini kit (Qiagen K.K.) in accordance with the manufacturer’s instructions.

### Blood and tissue collection

Whole blood was collected from mice at the age of 43 wk in the non-fasting condition via the post-caval vein under anesthesia with inhaled sevoflurane (SEVOFRAN®; Maruishi Pharmaceutical Co., Ltd., Osaka, Japan). The blood was then immediately analyzed. The remaining blood was maintained at 4ºC until plasma preparation. Immediately after euthanasia by exsanguination, the quadriceps, gastrocnemius, plantaris, extensor digitorum longus (EDL), and soleus muscles, along with the epididymal, perirenal, and retroperitoneal white adipose tissues (WAT) and the liver, were removed and weighed. Tissue samples were stored at –80ºC until analysis.

### Biochemical analysis

The concentrations of erythrocytes, leukocytes, and platelets, along with hemoglobin concentration and the hematocrit, were measured in heparinized blood with an automatic hematocytometer (Celltac MEK-5258; Nihon Kohden, Tokyo, Japan). Plasma was obtained from the blood by centrifugation at 3500 × *g* for 15 min. Plasma glucose; triglycerides (TG); non-esterified fatty acid (NEFAs); aspartate aminotransferases (AST); alanine aminotransferase (ALT); total-cholesterol; lactate; lactate dehydrogenase (LDH); and ketone bodies were quantified by using N-A Glu-UL, N-A L TG-H, NEFA-HA, N-A L GOT, N-A L GPT, N-A L T-CHO-H, N-A L LAC, N-A L LDH, and T-KB-H assay kits (Nittobo Medical Co., Ltd., Tokyo, Japan), respectively. Plasma insulin-like growth factor (IGF)-1 was measured with a mouse IGF-1 immunoassay (R&D Systems Inc., Minneapolis, MN). Plasma adiponectin levels were measured with a mouse/rat adiponectin ELISA kit (Otsuka Pharmaceuticals Co. Ltd, Tokyo, Japan). All measurements were performed in accordance with the manufacturers’ instructions.

### Force of soleus and EDL muscle contractions induced by electrical stimulation

Muscle force measurements were performed as described previously (Haramizu et al. 2011a, 2011b). The muscle of the right leg was quickly isolated. The muscle was anchored horizontally between two hooks–one fixed and one attached to an isometric force transducer (World Precision Instruments, Inc., Sarasota, FL)–and immersed in Krebs solution of the following composition: 119.7 mM NaCl, 4.5 mM KCl, 0.5 mM MgCl_2_, 0.7 mM Na_2_HPO_4_, 1.5 mM NaH_2_PO_4_, 15 mM NaHCO_3_, 2.5 mM CaCl_2_, and 10 mM D-glucose; pH 7.3 ± 0.1. The solution was continuously bubbled with 95% O_2_ – 5% CO_2_ at 37ºC. The muscle was electrically stimulated with a stimulus-isolation unit (SEN-3301; Nihon Kohden, Japan) and the optimal twitch length was set. Twitch force was measured with a single pulse; tetanic responses were induced with a 0.2-ms pulse (140 Hz) for 330 ms once every 2 s and digitally recorded for 2 min with a bridge amplifier and data acquisition system (Quad-16I; World Precision Instruments, Inc.). Measurements were analyzed with Data-Trax software (World Precision Instruments, Inc.).

### Indirect calorimetry

To elucidate the effect of MFGM or GTE with or without exercise on energy metabolism, we measured the oxygen consumption (VO_2_) and respiratory quotient (RQ) of the mice after 17 to 18 wk of feeding. We used an indirect calorimetric system equipped with a sixteen-chamber airtight metabolic cage (ARCO2000-RAT/ANI 16 Chamber System®; Arcosystem Inc., Chiba, Japan) (Murase et al. 2011). Each mouse was placed in a chamber for 3 d and allowed to acclimate to the surroundings before the measurement. Oxygen consumption and carbon dioxide production were then measured under feeding conditions for 24 h. RQ was calculated by dividing the measured values of carbon dioxide production by those of oxygen consumption. During the measurement, locomotor activity was measured with an automated motion analysis system (Actracer2000; Arcosystem Inc.), which detects the amount of centroid fluctuation by using a weighted transducer.

### DNA microarray analysis

Total RNA was extracted from frozen quadriceps muscle (n = 4) by using an RNeasy Fibrous Tissue Mini kit (Qiagen K.K., Tokyo, Japan) in accordance with the manufacturer’s instructions. For microarray analysis, the quality of total RNA samples was checked with an Agilent 2100 BioAnalyzer (Agilent Technologies Inc, Tokyo, Japan); the RNA integrity number of each sample was over 7.0. DNA microarray analysis was performed with a one-color system and the Agilent Mouse SurePrint G3 mouse gene expression array. In brief, 200 ng of each RNA was labeled and amplified with a Low Input Quick Amp Labeling Kit (one color; Agilent Technologies) in accordance with manufacturer’s instructions. Cyanine 3-labeled cRNA was fragmented and hybridized by using a Gene Expression Hybridization Kit and then washed with a Gene Expression Wash Pack (both Agilent Technologies). The hybridized microarray slides were scanned with an Agilent Technologies Microarray Scanner (Agilent Technologies) and the data were extracted by using Agilent Feature Extraction 10.7.1 in Hokkaido-System Science (Sapporo, Japan). After we had confirmed the high degree of reliability of the microarray processes on the basis of the QC report, data normalization and filtering were performed with GeneSpring GX 11.5 (Agilent technologies), as follows: 1) threshold raw signals were set to 1.0.; 2) 75th percentile normalization was used for the normalization algorithm; 3) the baseline was transformed to the median of all samples; and 4) raw values filtered by signal intensity value (upper cut-off: 100^th^ percentile; lower cut-off: 20^th^ percentile) of raw values and flagging to exclude absent reads. Probe sets were then identified by using unpaired *t*-test (*P* < 0.05) to compare between the experimental and control SAMP1 groups, with a relative fold-change value of >1.2 and no correction for multiple testing; this was done because the MicroArray Quality Control Consortium suggests the use of a fold-change cut off along with a non-stringent *P*-value cut-off as a baseline practice to improve reproducibility in microarray data processing (MAQC Consortium et al. 2006). Selected probe sets were analyzed with IPA (Ingenuity Pathways Analysis) software version 9.0 (Ingenuity Systems, Redwood City, CA).

### Quantitative real-time polymerase chain reaction (RT-PCR)

The microarray expression results were verified by using quantitative RT-PCR. The quadriceps muscles of mice in all groups (n = 7 or 8) were analyzed for gene expression as described previously (Haramizu et al. 2011a, 2011b). The following mouse-specific primer sequences were used for the mRNAs for the following proteins: docking protein (Dok)-7 forward, TGAGCTTCCTGTTTGACTGCA; Dok-7 reverse, GCAACACGCTCTTCTGAGGC; muscle skeletal receptor-tyrosine kinase (MuSK) forward, CATGGCAGAGTTTGACAACCC; MuSK reverse, TTCGGAGGAACTCATTGAGGTC; neural cell adhesion molecule (NCAM) forward, CAGTGACCACGTCATGCTCAAG; NCAM reverse, CCTGAACACAAAGTGAGCTGCC; myogenic differentiation (MyoD)-1; CTAGATCCAGCCCCAAAGAAAG, MyoD reverse, AGGTGCAGCCAGAGTGCAA; myogenin forward, GCACTGGAGTTCGGTCCCA; and myogenin reverse, GTGATGCTGTCCACGATGGA. For quantitative precision, the same amount of total RNA was consistently used for each expression analysis. Expression of each gene was normalized against that of the housekeeping gene encoding ribosomal protein, large, P0 (RPLP0/36B4).

### Western blot analysis

Quadriceps muscles were homogenized and lysed on ice in a Physcotron homogenizer (Microtech, Chiba, Japan) and a ready-made homogenization buffer, CelLytic MT Mammalian Tissue Lysis/Extraction Reagent (Sigma, St Louis, MO) containing a protease inhibitor cocktail (Sigma), and phosphatase inhibitor cocktail-1 and -2 (Sigma). After centrifugation of the muscle mixtures at 12,000 × *g* for 15 min at 4°C, the supernatants were removed and their protein concentrations determined with a BCA protein assay kit (Pierce, Rockford, IL). Equal amounts of protein (1 μg/μL) were boiled at 100°C for 5 min in SDS sample buffer (Novagen, Inc., Madison, WI) and centrifuged at 2400 × *g* for 5 min at 4°C. The protein extracts were separated by sodium dodecyl sulfate polyacrylamide gel electrophoresis (SDS-PAGE) and transferred to Immobilon-P polyvinylidene fluoride (PVDF) membranes (Millipore Corp., Bedford, MA) at 200 mA for 1.5 h. The membranes were then blocked with PVDF Blocking Reagent for Can Get Signal (Toyobo Co., Ltd., Osaka, Japan) at room temperature for 1 h and incubated overnight with anti-Dok-7 (1:1000 dilution, Abcam, Cambridge, UK) and anti-MuSK (R&D Systems Inc., Minneapolis, MN) or anti-α-tubulin (1:1000 dilution, Cell Signaling, Beverly, MA) primary antibodies in Immunoreaction Enhancer Solution 1 (Toyobo). After being washed four times with Tris-buffered saline containing 0.05% Tween 20 (Bio-Rad Laboratories, Hercules, CA), the membranes were incubated with horseradish-peroxidase-labeled anti-rabbit (Cell Signaling) immunoglobulin for Dok-7 and α-tubulin or anti-goat immunoglobulin for MuSK (Wako, Osaka, Japan) in Immunoreaction Enhancer Solution 2 (Toyobo). The blots were visualized with an ECL Prime Western Blotting Detection System (GE Healthcare, Buckinghamshire, UK) and a ChemiDoc XRS imaging system (Bio-Rad).

### Statistical analysis

All values are presented as means ± standard error (SE). Unpaired Student’s *t*-tests after a preliminary F-test of the homogeneity of within-group variance were used to compare values between groups. When more than two groups were compared, statistical analysis was conducted with one-way ANOVA followed by Fisher’s protected least significant difference or Dunnett’s post-hoc tests (Statview for Windows version 5.0, SAS Institute Inc., Cary, NC). A *P* value of less than 0.05 was considered statistically significant.

## Results

### Effect of exercise plus MFGM on body and tissue weights in SAMP1

Body and tissue weights of mice at the age of 43 wk are shown in Table 1. The quadriceps, gastrocnemius, plantaris, EDL and soleus muscles in the control SAMP1 group weighed significantly less than those in the ICR group. In contrast, the epididymal, perirenal, and retroperitoneal WAT tissues were significantly heavier in the control SAMP1 group than in the ICR group. The quadriceps muscle weighed significantly more in the MFGMEx mice than in the control SAMP1 group, whereas the weights of the gastrocnemius, plantaris, EDL and soleus muscles were similar between the groups. The GTE and GTEEx groups had significantly lower body weights than the control SAMP1 group. Fat and liver weights were significantly lower in both the GTE and the GTEEx group than in the control SAMP1, whereas muscle weight did not differ between the groups. Dietary intake was significantly higher in the ICR group than in the control SAMP1 group, but body weights did not differ between the ICR mice and the control SAMP1.

**Table 1:** **Body weight, feed intakes, feed efficiency, and tissues weights**

	ICR	Control	MFGM	MFGMEx	GTE	GTEEx
		SAMP1				
Body weight, g	44.2 ± 1.4	42.2 ± 2.7	42.2 ± 1.7	40.3 ± 1.6	29.6 ± 1.9^*^	32.5 ± 1.0^*^
Dietary intake, g	791.0 ± 26.2^*^	584.7 ± 13.9 ^‡‡^	564.1 ± 8.5	632.6 ± 24.8	527.5 ± 3.8	569.8 ± 4.1
Quadriceps, mg	424.6 ± 13.0^*^	318.0 ± 6.0 ^‡‡^	306.3 ± 6.4	355.8 ± 8.5^*^	301.1 ± 16.8	323.9 ± 11.5
Gastrocnemius, mg	328.8 ± 14.1^*^	228.8 ± 7.6 ^‡‡^	222.4 ± 5.5	234.3 ± 5.8	198.5 ± 12.3	238.2 ± 16.7
Plantaris, mg	44.5 ± 2.7^*^	27.3 ± 1.8 ^‡‡^	28.5 ± 2.6	29.5 ± 2.2	25.6 ± 2.5	27.8 ± 1.9
EDL, mg	27.6 ± 0.8^*^	20.4 ± 0.8 ^‡‡^	19.2 ± 1.4	19.9 ± 0.9	19.1 ± 0.4	18.4 ± 1.4
Soleus, mg	20.1 ± 1.3^*^	15.6 ± 0.8 ^†^	17.1 ± 0.8	17.1 ± 1.1	15.4 ± 1.3	16.6 ± 1.2
Epididymal fat, g	1.43 ± 0.13^*^	2.34 ± 0.22 ^††^	2.48 ± 0.15	2.31 ± 0.22	1.11 ± 0.31^*^	1.29 ± 0.25^*^
Perirenal fat, g	0.19 ± 0.03^*^	0.37 ± 0.07 ^†^	0.33 ± 0.05	0.26 ± 0.03	0.11 ± 0.03^*^	0.15 ± 0.02^*^
Retroperitoneal fat, g	0.37 ± 0.03	0.56 ± 0.07 ^†^	0.58 ± 0.05	0.46 ± 0.04	0.22 ± 0.06^*^	0.23 ± 0.06^*^
Liver, g	1.39 ± 0.08	1.36 ± 0.07	1.36 ± 0.04	1.27 ± 0.03	1.09 ± 0.03^*^	1.09 ± 0.03^*^

Values are means ± S.E. of 7 or 8 mice.

^†^ *P* < 0.05, ^††^ *P* < 0.01, ^†††^ *P* < 0.001,^‡‡^ *P* < 0.0001, significant difference between ICR group and control SAMP1 group at the age of 43 wk by unpaired *t*-test.

^*^ *P* < 0.05, significant difference vs. control SAMP1 group at the age of 43 wk by Dunnett’s test.

### Effect of exercise plus MFGM on blood and plasma components in SAMP1

The blood level of erythrocytes, as well as the hemoglobin, and hematocrit values, was significantly lower in the control SAMP1 group than in the ICR group at the age of 43 wk (Table 2). The hemoglobin and hematocrit values were significantly higher in the MFGMEx group than in the control SAMP1 group. We measured plasma adiponectin and IGF-1 levels, because they play important roles in regulating energy metabolism (Kahn et al. 2005) and protein synthesis in skeletal muscle (Perrini et al. 2010). The plasma adiponectin level was significantly lower (*P* < 0.05), and the plasma IGF-1 level tended to be lower (*P* = 0.07) in the control SAMP1 group than in the ICR group. The MFGMEx group had significantly higher plasma adiponectin and IGF-1 levels than did the control SAMP1 group. The control SAMP1 group had significantly lower plasma LDH and AST and significantly higher TG levels than did the ICR mice. These plasma measurements did not differ between the experimental groups and the control SAMP1 group.

**Table 2:** **Blood and plasma analysis**

	ICR	Control	MFGM	MFGMEx	GTE	GTEEx
		SAMP1				
Blood component						
WBC, 10^2^/μL	46.4 ± 13.6	38.7 ± 8.1	34.4 ± 4.7	28.5 ± 4.6	41.3 ± 13.7	32.8 ± 9.3
RBC, 10^4^/μL	844.4 ± 19.8^*^	730.1 ± 21.7^††^	771.6 ± 16.7	779.5 ± 19.7	675.0 ± 27.9	696.2 ± 55.6
Haemoglobin, mg/dL	13.8 ± 0.2^*^	12.3 ± 0.3^††^	12.7 ± 0.2	13.5 ± 0.3^*^	11.5 ± 0.4	12.3 ± 0.2
Haematocrit,%	43.2 ± 0.8^*^	37.0 ± 1.0^‡^	38.8 ± 0.8	40.3 ± 1.1^*^	35.1 ± 1.1	36.4 ± 2.1
Platelet, 10^4^/μL	93.0 ± 6.4	73.9 ± 2.8^†^	76.6 ± 3.4	72.8 ± 9.0	93.2 ± 11.2	72.6 ± 4.5
Plasma component						
Glucose, mg/dL	210.8 ± 18.5	230.8 ± 17.1	217.2 ± 5.7	207.7 ± 12.1	219.9 ± 26.7	241.2 ± 19.2
Lactate, mg/dL	55.0 ± 5.1	63.5 ± 4.0	56.2 ± 4.3	66.1 ± 7.6	68.0 ± 10.3	66.1 ± 11.3
LDH, IU/L	287.4 ± 34.8^*^	150.6 ± 13.7^††^	158. 7 ± 7.8	150.3 ± 11.8	171.3 ± 19.3	148.2 ± 9.2
AST, IU/L	48.5 ± 5.3^*^	28.4 ± 1.3^††^	28.9 ± 1.9	28.1 ± 2.7	37.3 ± 3.0	33.8 ± 2.0
ALT, IU/L	15.7 ± 0.8	16.8 ± 2.8	18.6 ± 1.8	19.2 ± 4.3	14.2 ± 2.1	16.1 ± 2.4
Total Chol, mg/dL	163.2 ± 20.9	170.7 ± 6.8	189.1 ± 13.5	188.9 ± 11.0	159.1 ± 13.9	160.2 ± 11.8
TG, mg/dL	55.8 ± 8.7	88.5 ± 7.4^†^	104.7 ± 19.4	114.4 ± 24.0	82.6 ± 11.7	90.1 ± 14.6
Ketone body, mg/dL	163.1 ± 41.0	248.8 ± 30.0	219.8 ± 45.5	242.6 ± 55.8	310.6 ± 79.7	329.4 ± 86.5
NEFA, mEq/L	0.9 ± 0.1	1.0 ± 0.1	1.0 ± 0.1	1.1 ± 0.1	0.8 ± 0.1	1.0 ± 0.1
Adiponectin, pg/mL	3.5 ± 0.2^*^	2.0 ± 0.1^‡‡^	2.0 ± 0.1	2.4 ± 0.1	2.0 ± 0.2	2.1 ± 0.1
IGF-1, ng/mL	596.3 ± 53.7^*^	474.2 ± 14.1	501.6 ± 10.4	549.2 ± 26.9	438.6 ± 24.8	426.8 ± 25.9

Values are means ± S.E. of 7 or 8 mice.

^†^ *P* < 0.05, ^††^ *P* < 0.01, ^‡‡^ *P* < 0.0001, significant difference between ICR group and control SAMP1 group at the age of 43 wk by unpaired *t*-test.

^*^ *P* < 0.05, Significant difference vs. control SAMP1 group at the age of 43 wk by Dunnett’s test.

### Effect of exercise plus MFGM on tetanic contractile force of soleus and EDL muscles in SAMP1

**Figure 1:**
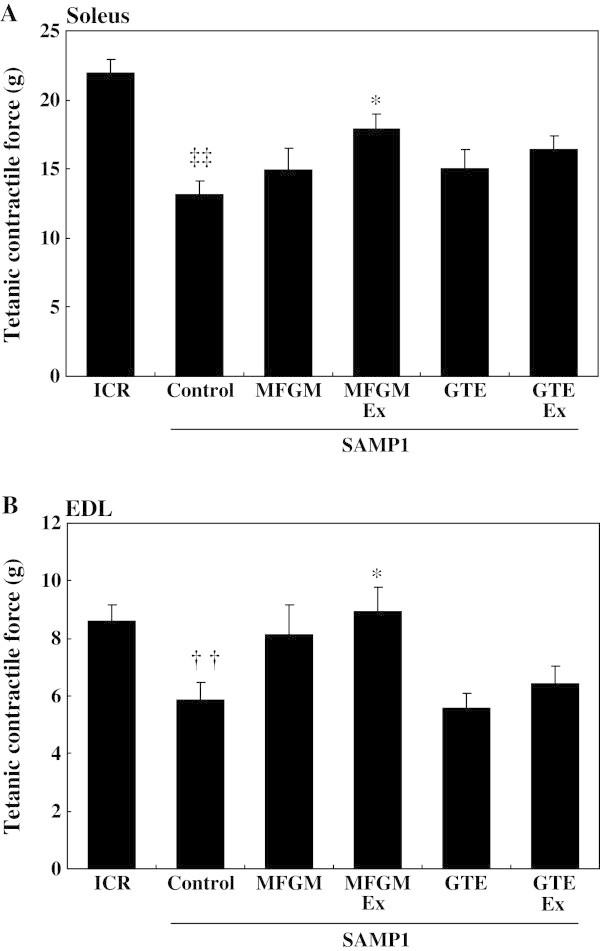
**Effects of milk-fat globule membrane (MFGM) or green tea extract (GTE) on tetanic contractile force of soleus and extensor digitorum longus (EDL) muscles.** The tetanic contractile force of the isolated soleus **(A)** and EDL **(B)** muscles was measured as described in the Methods. Values are means ± S.E. of 7 or 8 mice. ^††^ *P* < 0.01 and ^‡‡^ *P* < 0.0001, significant difference between ICR group and control SAMP1 group by unpaired *t*-test. ^*^ *P* < 0.05, significant difference vs. the control SAMP1 group by Dunnett’s posthoc test. MFGMEx, MFGM plus habitual exercise; GTEEx, GTE plus habitual exercise.

The tetanic contractile force of the soleus (Figure 1A) and EDL (Figure 1B) muscles was significantly lower in the control SAMP1 group than in the ICR group at the age of 43 wk. The MFGMEx group had significantly higher tetanic contractile force than the control SAMP1 group in the soleus (Figure 1A) and EDL (Figure 1B) muscles. Contractile force in the GTEEx group did not differ significantly from that in the control SAMP1 group.

### Effect of exercise plus MFGM on locomotor activity and energy metabolism in SAMP1

Spontaneous locomotor activity and VO_2_ were significantly lower in the control SAMP1 group than in the ICR group at the age of 40 – 41 wk (*P* < 0.05; Table 3). In contrast, the MFGMEx group had significantly higher spontaneous locomotor activity and VO_2_ than did the control SAMP1 group (*P* < 0.05). Oxygen consumption did not differ between GTE-fed mice (GTE and GTEEx groups) and the control SAMP1 mice. There were no differences in RQ between the experimental groups.

**Table 3:** **Energy metabolism and spontaneous activity**

	ICR	Control	MFGM	MFGMEx	GTE	GTEEx
		SAMP1				
Activity, g*cm/min	154.11 ± 18.69	92.02 ± 16.75^†^	115.99 ± 15.51	171.21 ± 25.17^*^	72.28 ± 9.45	135.36 ± 16.34
VO_2_, ml/min	2.48 ± 0.09^*^	2.10 ± 0.10^†^	2.18 ± 0.09	2.45 ± 0.06^*^	1.82 ± 0.10	2.03 ± 0.07
RQ	0.851 ± 0.028	0.834 ± 0.026	0.835 ± 0.021	0.855 ± 0.022	0.838 ± 0.024	0.794 ± 0.016

Values are means ± S.E. of 7 or 8 mice.

^†^ *P* < 0.05, significant difference between ICR group and control SAMP1 group at the age of 40 - 41 wk by unpaired *t*-test.

^*^ *P* < 0.05, significant difference vs. control SAMP1 group at the age of 43 wk by Dunnett’s test.

### Effects of exercise plus MFGM on muscle gene expression

To identify the potential molecular mechanisms underlying the beneficial effects of habitual exercise combined with dietary MFGM on muscle mass and force, we compared the transcriptomic profile in the quadriceps muscle between the MFGMEx and the control SAMP1 groups. In the microarray analyses, probe sets identified as differentially expressed between the groups had a relative fold change value of >1.2 with no correction for multiple testing. Volcano plot analysis in GeneSpring revealed that 893 probes were differentially expressed in the MFGMEx group compared with the control SAMP1 group. Among these 893 probes, 317 were identified as up-regulated and 576 probes were down-regulated (Additional file 1: Table S1). IPA was then used to decipher the biological processes characterized by the list differentially expressed probes. The biological processes highly represented in the MFGMEx group compared with the control SAMP1 group were “nervous system development and function” (*P* = 3.35E-05 to 2.25E-02), “hematological system development and function” (*P* = 1.59E-04 to 2.30E-02), “immune cell trafficking” (*P* = 1.59E-04 to 2.30E-02), “lymphoid tissue structure and development” (*P* = 1.59E-04 to 2.25E-02), and “embryonic development” (*P* = 5.04E-04 to 2.25E-02) within the hierarchy “physiological systems development and functions.” The top five functional annotations in the most significant process are shown in Table 4. The most significant function responding to the combination of habitual exercise and dietary MFGM was “formation of synapse,” followed by “growth of neurites” and “development of neuromuscular junction.” In contrast, the microarray analysis followed by the IPA analysis of the GTEEx and control SAMP1 groups showed that the most significant function responding to the combination of habitual exercise and dietary GTE was “hematological system development and function.” These functions by habitual exercise and dietary MFGM were not observed in response to combination of habitual exercise and dietary GTE (data not shown).

**Table 4:** “**Nervous System Development and Function” characterised by differentilly expressed probes in the quadriceps muscles of the control and MFGMEx groups, as determined by using IPA (Ingenuity Pathways Analysis) software v9.0**

Functional annotation	Differentially expressed genes/total genes	***P***-value
Formation of synapse	8/179	3.33E-05
Growth of neurites	18/708	1.89E-03
Development of neuromuscular junction	4/37	2.54E-03
Outgrowth of neurites	15/614	6.53E-03
Morphogenesis of neurites	7/438	1.58E-02

### Effects of exercise plus MFGM on expression of genes encoding IGF-1 signaling molecules

**Figure 2:**
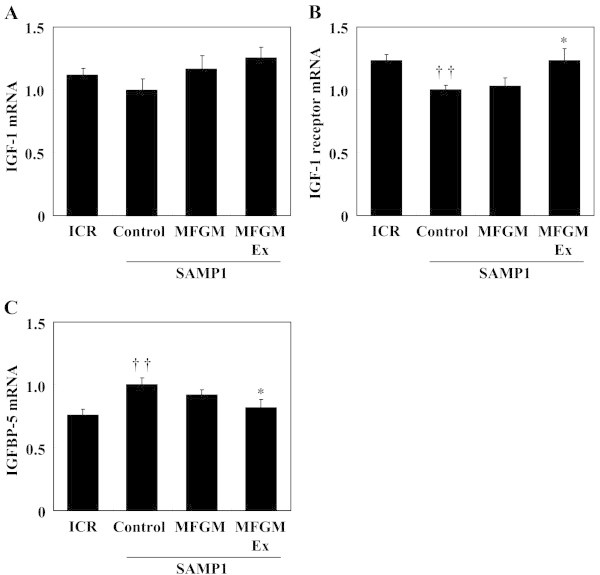
**Effects of milk-fat globule membrane (MFGM) on expression of genes associated with insulin-like growth factor (IGF)-1 signaling-related molecules.** mRNA expression levels of IGF-1 **(A)**, IGF-1 receptor **(B)**, and IGF-binding protein (IGFBP)-5 **(C)** were measured using quantitative real-time PCR. Values are means ± S.E. of 7 or 8 mice. ^††^ *P* < 0.01, significant difference between ICR group and control SAMP1 group by unpaired *t*-test. ^*^ *P* < 0.05, significant difference vs. control SAMP1 group by Fisher’s PLSD posthoc test. Values are expressed as ratios, using the value of the control SAMP1 group as 1.0. MFGMEx, MFGM plus habitual exercise.

To explore the differences in muscle weight between the SAMP1 controls and the ICR controls and (in the case of the quadriceps) between the MFGMEx group and the SAMP1 controls, the genes involved in muscle development were assessed by using RT-PCR. The control SAMP1 group had significantly lower *igf1*r and higher IGF-1 binding protein (*igfbp*)-*5* gene expression (18.8% and 32.2%, respectively) than the ICR group. IGF-1 mRNA expression level did not differ among the groups (Figure 2). The MFGMEx group had significantly higher *igf1r* and lower *igfbp5* gene expression (by 23.4% and 24.3%, respectively) than did the control SAMP1 group.

### Effect of exercise plus MFGM on MuSK and Dok-7

In light of the microarray results, the genes involved in “nervous system development and function” were examined. Microarray analysis revealed that habitual exercise plus dietary MFGM significantly increased MuSK mRNA expression (Additional file 1: Table S1). Because MuSK and Dok-7 are crucial for NMJ formation (DeChiara et al. 1996; Inoue et al. 2009; Okada et al. 2006), we measured Dok-7 mRNA expression; our microarray analysis found only one instance in which the gene tended to be upregulated.

**Figure 3:**
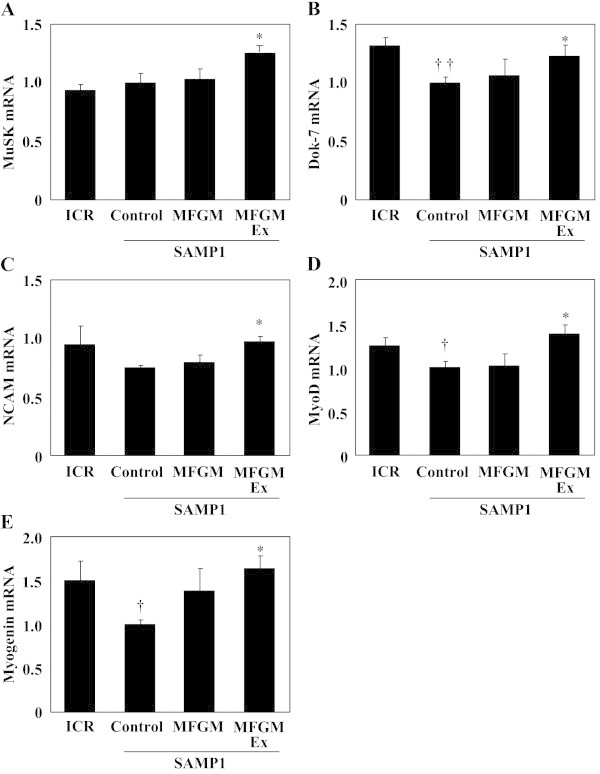
**Effects of milk-fat globule membrane (MFGM) on expression of genes associated with nervous system development and function-related molecules.** mRNA expression levels of MuSK **(A)**, Dok-7 **(B)**, NCAM **(C)**, MyoD **(D)**, and myogenin **(E)** were measured using quantitative real-time PCR. Expression of each gene was normalized against that of the housekeeping gene encoding ribosomal protein, large, P0 (RPLP0/36B4). Values are means ± S.E. of 7 or 8 mice. ^††^ *P* < 0.01 and ^†^ *P* < 0.05, significant difference between ICR group and control SAMP1 group by unpaired *t*-test. ^*^ *P* < 0.05, significant difference vs. control SAMP1 group by Fisher’s PLSD posthoc test. Values are expressed as ratios, using the value of the control SAMP1 group as 1.0. MuSK, muscle skeletal receptor-tyrosine kinase; Dok-7, docking protein-7; NCAM, neural cell adhesion molecule; MyoD, myogenic differentiation; MFGMEx, MFGM plus habitual exercise.

Expression of mRNAs for MyoD (+37.7%) and myogenin (+63.6%) was significantly higher in the MFGMEx group than in the control SAMP1 group (Figure 3), whereas it was significantly lower in the control SAMP1 group than in the ICR group. The MFGMEx group had significantly higher MuSK mRNA expression (+26.9%) than the control SAMP1 group, whereas MuSK mRNA expression did not differ between the ICR group and the control SAMP1 group. The MFGMEx group had significantly higher NCAM mRNA expression (+29.5%) than did the control SAMP1 group; these results were consistent with the microarray results. Whereas Dok-7 was not found in the microarray results (Additional file 1: Table S1), our RT-PCR analysis showed that the MFGMEx group had significantly greater Dok-7 (+31.0%) mRNA expression than the control SAMP1 group (Figure 3).

**Figure 4:**
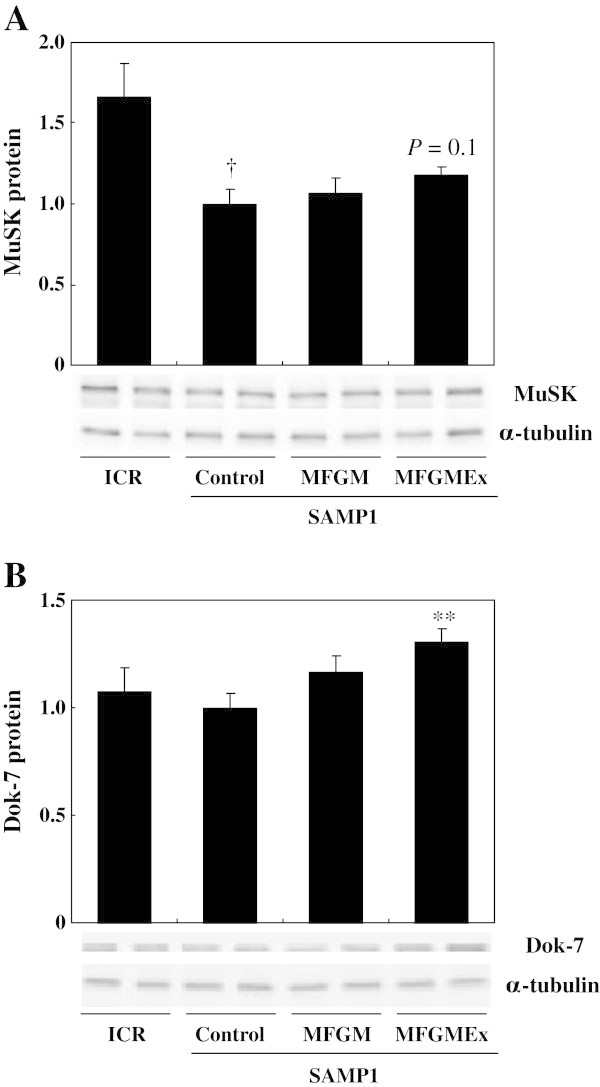
**Effects of milk-fat globule membrane (MFGM) on production of MuSK and Dok-7 proteins.** Levels of production of the proteins MuSK **(A)**, and Dok-7 **(B)** were measured by western blot analysis. The quadriceps muscle was removed from each mouse in the non-fasting, resting condition. Tissue lysates were then prepared and subjected to western blot analysis. Values are means ± S.E. of 7 or 8 mice. ^†^ *P* < 0.05, significant difference between ICR group and control SAMP1 group by unpaired *t*-test. ^**^ *P* < 0.01, significant difference vs. Control SAMP1 group by Fisher’s PLSD posthoc test. Values are expressed as ratios, using the value of the control SAMP1 group as 1.0. MFGMEx, MFGM plus habitual exercise.

The Dok-7 protein level was significantly higher (+30.8%, *P* < 0.01), and the MuSK protein level tended to be higher (+18.2%, *P* = 0.1), in the MFGMEx group than in the control SAMP1 group (Figure 4). ICR mice had significantly more MuSK protein than the control SAMP1 mice. The Dok-7 protein level did not differ between these two groups.

### Effects of MFGM, PLF, SLF, and sphingomyelin plus mechanical stretching on expression of genes encoding Dok-7, MuSK, and myogenin in differentiating C2C12 cells

**Figure 5:**
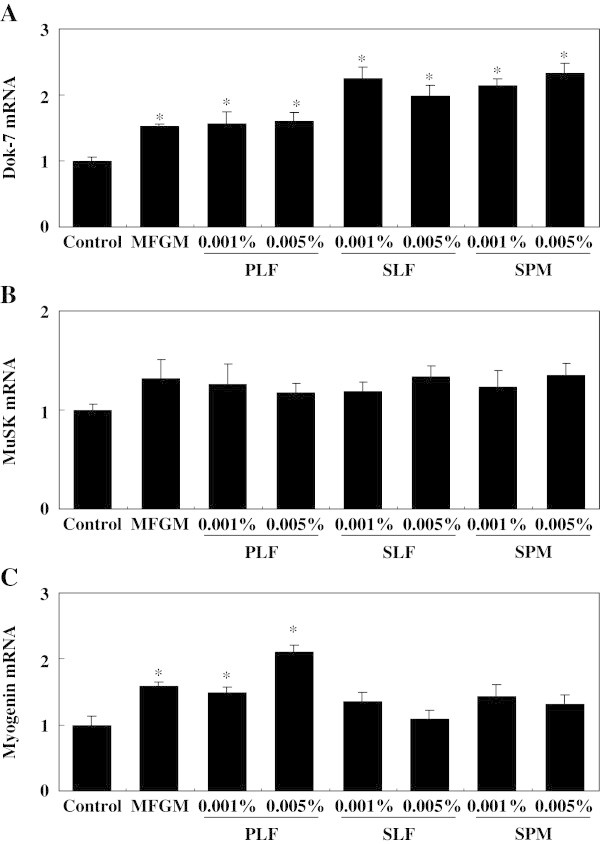
**Effects of milk-fat globule membrane (MFGM), the phospholipid fraction (PLF), the sphingolipid fraction (SLF), and sphingomyelin (SPM), plus mechanical stretching, on expression of the mRNAs of Dok-7, MuSK, and myogenin in differentiating C2C12 cells.** Levels of expression of the mRNAs of Dok-7 **(A)**, MuSK **(B)**, and myogenin **(C)** were measured by using quantitative real-time PCR. Expression of each gene was normalized against that of the housekeeping gene encoding ribosomal protein, large, P0 (RPLP0/36B4). Values are means ± S.E. of 6 samples. ^*^ *P* < 0.05, significant difference vs. control group by Dunnett’s posthoc test. Values are expressed as ratios, using the value of the control group as 1.0.

Skeletal muscle is highly adaptable and responds to exercise and training, thus increasing muscle mass and function. To further understand the mechanisms underlying the adaptive response of skeletal muscles in vivo, in vitro culture systems that use mechanical stretching of cultured myotubes have been developed to mimic in vivo muscle physiology (Passey et al. 2011), including neuromuscular adaptation in response to exercise (Folland and Williams 2007; Hubatsch and Jasmin 1997; Jasmin et al. 1991; Sveistrup et al. 1995). Accordingly, we examined the effects of MFGM-derived fractions and sphingomyelin combined with mechanical stretch (as a substitute for exercise in vivo) on the expression of several genes.Treatment of the stretched cells with MFGM, PLF, SLF, or sphingomyelin significantly increased Dok-7 gene expression compared with that in cells that received mechanical stretching alone; this was evident especially in the cells treated with SLF or sphingomyelin (Figure 5). Myogenin gene expression was increased significantly by treatment with 0.01% MFGM or with 0.001% or 0.005% PLF fraction plus mechanical stretching of the cells. These results are consistent with the effects of the combination of habitual exercise and dietary MFGM observed in vivo.

## Discussion

Our major finding was that habitual exercise combined with nutritional supplementation in the form of dietary MFGM, but not exercise plus dietary GTE, attenuated age-related loss of muscle mass and maximum contractile force in SAMP1; this result may have been due to the increase in metabolic rate and physical activity. Our results also suggest that the beneficial effects of habitual exercise plus dietary MFGM on skeletal muscle mass and function are related to the stimulation of neuromuscular system development and function.

The control SAMP1 group had smaller muscle mass and contractile force production than did ICR mice. These results were consistent with those of Sakakima et al. (2004) and with our previous finding that contractile force in the soleus muscle was significantly lower in SAMP1 mice than in SAMR1 mice (Haramizu et al. 2011a, 2011b). We used ICR mice as normal group of controls; our results suggest that, in comparison with these mice, SAMP1 mice exhibit aging-related deterioration in the mass and function of both soleus and EDL muscle. SAMP1 mice were less active either locomotively or metabolically; this may have been the cause of the increase in WAT accumulation and plasma TG levels, and the decrease in adiponectin levels, compared with those in ICR mice.

Even though we consider ICR mice to be valid controls for SAMP1 mice (Lee et al. 2013; Nagano et al. 2000; Sakakima et al. 2004), we cannot rule out the possibility that the observed differences in the results obtained with ICR and SAMP1 mice may be due to differences between the strains. Because Derave et al. (2005) stated that SAMR1 mice were not suitable because of difficulties in breeding them, and because we have had problems with tumors of the oral cavity in SAMR1 mice, we prefer to use ICR mice as controls for SAMP1 mice in our studies.

Our SAMP1 mice had significantly lower skeletal muscle mass than did ICR mice at age 43 wks. Considering the fact that there is no difference in the ratio of gastrocnemius muscle weight to body weight between ICR and SAMP1 mice at the age of 24 wk (Sakakima et al. 2004), the lower muscle mass in SAMP1 in this study is not likely to be caused by arrested muscle growth but by sarcopenia. Our previous study showed that the reduced muscle mass in SAMP1 could be explained partly by a decrease in IGF-1 signaling, which dynamically regulates muscle protein synthesis (Haramizu et al. 2011a, 2011b). IGF-1 receptor gene deletion in muscle causes earlier postnatal diabetes and mortality (Kitamura et al. 2003). Expression of the gene encoding IGFBP (binding protein)-5, decreases with age (Welle et al. 2001); IGFBP-5 is produced by muscle cells and suppresses muscle differentiation by interfering with IGF-1-dependent signaling (Mukherjee et al. 2008). We found that decreased expression of the mRNA for IGF-1 receptor and increased expression of the mRNA for IGFBP-5 mRNA expression, either of which can interfere with IGF-1 signaling, seemed to be associated with decreased mass of the quadriceps muscle in SAMP1.

Most interestingly, the aging-associated deteriorations in muscle mass and force were significantly attenuated by a combination of habitual exercise and dietary MFGM. Aging-dependent declines in serum IGF-1 levels may also contribute to loss of muscle mass (Perrini et al. 2010); a recent study has shown that loss of muscle mass in aged mice can be attenuated partly by increasing serum IGF-1 levels by feeding royal jelly (Niu et al. 2013). Therefore, the increases in serum IGF-1 and IGF-1 receptor mRNA levels and decreases in IGFBP-5 mRNA levels after habitual exercise plus dietary MFGM might have contributed to the increase in muscle mass and contractile force production in SAMP1.

SAMP1 had lower concentrations of circulating erythrocytes, and lower hemoglobin and hematocrit levels than did ICR mice, in parallel with previous findings that with advancing age these levels decline (Boggs and Patrene 1986; Coppola et al. 2000). Concurrently, the decreased erythrocytes and hemoglobin levels reduce the oxygen-carrying capacity of the blood (Tsai et al. 2010); this may be consistent with the lower oxygen consumption in SAMP1 in the present study. MFGM intake combined with exercise increased hemoglobin and hematocrit levels and tended to increase the RBC count in the SAMP1 mice; this may have resulted in increased oxygen consumption. The protective effect of habitual exercise plus dietary MFGM on erythrocytes (including their survival, synthesis, and degradation) needs to be elucidated. Here, we did not measure mitochondrial and oxidative enzyme activities. In our previous studies, the lower physical performance of SAMP1 mice has been associated with a decrease in muscle β-oxidation capacity and in the mRNA expression levels of cytochrome c oxidase and peroxisome proliferator–activated receptor-gamma coactivator-1 (Haramizu et al. 2011a, 2011b; Murase et al. 2008). Therefore, we cannot rule out the possibility that a decrease in muscle mitochondrial activity for processing oxygen is also responsible for the lower whole-body oxygen consumption in SAMP1 mice.

Plasma adiponectin levels were lower in SAMP1 than in ICR mice but increased after MFGM intake plus habitual exercise. Adiponectin stimulates energy metabolism in skeletal muscle through the action of AMP-activated protein kinase (Kahn et al. 2005), and the absence of adiponectin causes muscle dysfunction (Krause et al. 2008). Therefore, our results suggest that an increase in plasma adiponectin levels also contributes to the increase in muscle force and whole-body energy expenditure seen with MFGM intake plus exercise. Bouassida et al. (2010) have shown that both acute and regular exercise increase circulating adiponectin levels. However, increase in adiponectin levels was observed by exercise plus GTE. Therefore, the results seem to be related more to the effect of exercise plus MFGM than of exercise alone.

Of more interest is our finding that the major effect of habitual exercise plus dietary MFGM was characterized in our transcriptomic analysis as ‘nervous system development.’ A combination of habitual exercise and dietary MFGM increased the levels of MuSK and Dok-7, both of which play essential roles in synapse formation at the NMJ because lacking MuSK or Dok-7 failed to form NMJ formation (DeChiara et al. 1996; Inoue et al. 2009; Okada et al. 2006), suggesting that exercise plus MFGM may help to improve NMJ formation. A combination of habitual exercise and dietary MFGM also increased the levels of expression of the mRNAs for MyoD and myogenin. The increased levels of expression of the mRNAs for MyoD and myogenin after exercise plus MFGM may help to improve NMJ formation and thereby improve muscle contractile function, because lack of MyoD expression results in aberrant development of neuromuscular synapses, leading to muscle contractile dysfunction (Macharia et al. 2010). In addition, MyoD and myogenin are associated with muscle differentiation (Capkovic et al. 2008). Therefore, increased expression of the genes encoding MyoD and myogenin may also contribute to increased neural or muscle adaptation (i.e., through an in the number of myonuclei), or both, after habitual exercise plus dietary MFGM.

In contrast to our findings, some studies have found that the expression of some genes (e.g., encoding those encoding myogenin and NCAM) increases in denervation or aging (Ibebunjo et al. 2013; Larkin et al. 2003; Moresi et al. 2010). Although we cannot dismiss the difference between our findings and these previous ones, it is possible that the muscle of our SAMP1 at the age of 43 wk, unlike those in these previous models of severe aging and denervation, did not in fact undergo severe denervation. Therefore, the decreased levels of mRNAs for myogenin and NCAM in our SAMP1 mice might not have been associated with muscle denervation. Similarly, considering our finding that quadriceps muscle mass and soleus force was significantly increased by exercise plus MFGM in SAMP1, the increased levels of myogenin and NCAM mRNA expression seen with exercise plus MFGM do not suggest the presence of muscle denervation. However, more detailed analyses, for example by histological and molecular approaches, are needed to translate the transcriptional changes that we found here into improved muscle mass, function, and metabolism after a combination of habitual exercise and daily consumption of MFGM.

Our study had some limitations. First, since we did not count the rotations of the running wheel consistently during the experimental period, we cannot rule out the possibility that a combination of habitual exercise plus dietary MFGM increased total physical activity and thus affected muscle physiology. However, our preliminary study showed that dietary supplementation with 1% MFGM did not change spontaneous activity in mice (data not shown). Therefore, we speculate that the beneficial effects of exercise plus MFGM are not the result of a change in spontaneous activity levels.

Second, because we did not include a group subjected to habitual exercise alone, the effects on muscle mass, function, and NMJ formation may have been the result of exercise alone. A numbers of studies have shown that voluntary exercise alone in an unloaded condition (treadmill running and spontaneous wheel running) fails to improve muscle mass and strength (Ishihara et al. 1998; Gallo et al. 2006; Legerlotz et al. 2008); considering that resistance training is effective in improving muscle mass and strength (Chalé et al. 2013; Leenders et al. 2013), voluntary exercise may be difficult in managing muscle mass and strength when used alone. Consistent with this point, habitual running alone and dietary supplementation with green tea catechins combined with habitual running did not improve muscle mass (Murase et al. 2008). Here, habitual exercise plus dietary GTE did not change either muscle mass, or function, or the expression of NMJ-associated genes. In addition, the combination of mechanical stretch plus MFGM supplementation, but not mechanical stretch alone, increased the expression of genes involved in muscle differentiation and NMJ formation (Figure 5); this was consistent with the effects of habitual exercise plus dietary MFGM in vivo. Taking the results together enables us to conclude that the beneficial effects of the combination of habitual exercise and dietary MFGM are produced by the interaction between habitual exercise and dietary MFGM, and are unlikely to be produced by habitual exercise alone. Nevertheless, further studies are required to clarify the mechanism underlying these effects of combined habitual exercise and dietary MFGM.

Consumption of whole milk by young adults after resistance training promotes muscle protein synthesis and inhibits protein breakdown, leading to improved net muscle protein balance (Elliot et al. 2006; Josse et al. 2010; Wilkinson et al. 2007). Moreover, a cohort study has revealed that muscle strength in community-dwelling elderly is affected by the type of milk feeding in infancy (Robinson et al. 2012). The beneficial effects of milk on muscle mass and function are thought to be due to its nutritional capacity as a good source of proteins, lipids, amino acids, vitamins, and minerals. Cantó et al. (2012) have shown that nicotinamide riboside (the NAD^+^ precursor found in milk) activates sirtuin activity, enhances mitochondrial gene expression, and prevents diet-induced obesity. We demonstrated here that MFGM may also be a beneficial component of milk that, when combined with habitual exercise, suppresses aging-associated deterioration of muscle mass and strength and loss of NMJ formation. Studies are in progress to clarify the clinical efficacy of dietary supplementation with MFGM combined with regular exercise in human adults including the elderly.

We found here that while myogenin mRNA expression was upregulated by MFGM-derived phospholipids, Dok-7 mRNA expression was upregulated by MFGM-derived phospholipids and sphingolipids, and by milk-derived sphingomyelin in differentiating myoblasts under mechanical stretch, suggesting that the beneficial effects of habitual exercise plus dietary MFGM in vivo are at least partly due to these components. Lipids play crucial roles in various cellular functions. For example, phosphatidylserine is involved in myoblast fusion (van den Eijnde et al. 2001) and phosphatidylcholine triggers IGF-1-stimulated responses (Rauch and Loughna 2005). Moreover, sphingomyelin levels in the plasma membrane, a reservoir of bioactive sphingolipids, decrease during muscle satellite cell activation (Nagata et al. 2006), and increased levels of sphingosine-1-phosphate and sphingosine (metabolites of sphingomyelin) individually attenuate fatigue-induced decline in muscle contractile force (Danieli-Betto et al. 2005). In our preliminary experiment in rats, MFGM ingestion increased the contents of phospholipids, sphingolipids, free fatty acids, and triglycerides in the mesenteric lymph, suggesting that dietary MFGM is absorbed and circulates after being metabolized into phospholipids and sphingolipids (our unpublished observation). Therefore, we speculate that phospholipids and sphingolipids contribute to a mechanism by which MFGM combined with habitual exercise improves muscle function. However, further studies are required to elucidate the effects of this combination in vivo.

## Conclusions

Our findings provide evidence that, in senescence-accelerated mice, a combination of habitual exercise and dietary supplementation with MFGM improves age-related deficits in muscle function by improving neuromuscular development and IGF-1 signaling. Further studies are needed to clarify the mechanism underlying the interaction between regular exercise and dietary MFGM.

## Authors’ information

Satoshi Haramizu is a research scientist at the R&D - Biological Science Research of Kao Corporation. Mr. Haramizu’s research focus is nutritional and exercise physiology for health.

Takuya Mori is a research scientist at the R&D - Biological Science Research of Kao Corporation. Mr. Mori’s research focus is on nutritional physiology for health.

Michiko Yano, PhD, is a research scientist at the R&D - Biological Science Research of Kao Corporation. Dr. Yano’s research focus is on muscle biology for health.

Noriyasu Ota, is a principal research scientist at the R&D - Biological Science Research of Kao Corporation. Mr. Ota’s research focus is on nutritional and exercise physiology for health.

Kohjiro Hashizume is a research scientist at the R&D - Biological Science Research of Kao Corporation. Mr. Hashizume’s research focus is on organic chemistry for finding beneficial food compositions.

Atsuko Otsuka is a research scientist at the R&D - Biological Science Research of Kao Corporation. Mrs. Otsuka’s research focus is on muscle biology for health.

Tadashi Hase DVM is a Vice-President with responsibility for the R&D - Biological Science Research of Kao Corporation. Dr. Hase’s research focus is on lipid metabolism, nutrition, and health science.

Akira Shimotoyodome, PhD, is a Director for the R&D - Biological Science Research of Kao Corporation. Dr. Shimotoyodome’s research focus is on lipid metabolism, nutrition, and health science.

## Electronic supplementary material

Additional file 1: Table S1: Probe names that were increased or decreased by MFGM intake combined with exercise in the quadriceps muscle. (DOC 177 KB)

## Abbreviations

MFGM: Milk fat globule membrane (MFGM)
GTE: Green tea extract
SAMP1: Senescence-accelerated P1 mice
Dok-7: Docking protein-7
NMJ: Neuromuscular junction
SAMR: Senescence-accelerated resistant mouse
PLF: Phospholipid fraction
TLC: Thin-layer chromatography
SLF: Sphingolipid fraction
DMEM: Dulbecco’s modified eagle medium
EDL: Extensor digitorum longus
WAT: White adipose tissues
TG: Triglycerides
NEFA: Non-esterified fatty acid
AST: Aspartate aminotransferases
ALT: Alanine aminotransferase
HDL: High-density lipoprotein
LDL: Low-density lipoprotein
LDH: Lactate dehydrogenase
IGF: Insulin-like growth factor
VO_2_: Oxygen consumption
RQ: Respiratory quotient
MuSK: Muscle skeletal receptor-tyrosine kinase
NCAM: Neural cell adhesion molecule
MyoD: Myogenic differentiation
RPLP0/36B4: Ribosomal protein, large, P0
PVDF: Immobilon-P polyvinylidene fluoride
SDS-page: Sodium dodecyl sulfate polyacrylamide gel electrophoresis
SE: Standard error
ANOVA: Analysis of variance
IGFBP: IGF-binding protein.
