# Supplementary material for: Habitual exercise plus dietary supplementation with milk fat globule membrane improves muscle function deficits via neuromuscular development in senescence-accelerated mice
Source: Springerplus. 2014 Jul 4;3:339. doi: 10.1186/2193-1801-3-339 (PMC4125610; doi:10.1186/2193-1801-3-339)
Supplement: Supplementary file 1 — Additional file 1: Table S1: Probe names that were increased or decreased by MFGM intake combined with exercise in the quadriceps muscle. (DOC 177 KB) [file 40064_2014_1092_MOESM1_ESM.doc]

Additional file 1: Table S1. Probe names that were increased or decreased by MFGM intake combined with exercise in the quadriceps muscle

Probe name Gene symbol Fold change Description

Increased

A_52_P413395 Sln 4.391 Mus musculus sarcolipin (Sln), mRNA [NM_025540]

A_66_P114804 Gm7325 3.463 PREDICTED: Mus musculus predicted gene, EG653016 (EG653016), mRNA

[XM_001000918]

A_55_P2049717 Agrp 3.185 Mus musculus agouti related protein (Agrp), mRNA [NM_007427]

A_55_P2078695 Fbxl13 2.617 Mus musculus F-box and leucine-rich repeat protein 13 (Fbxl13), mRNA [NM_177076]

A_55_P2065562 Aldh3a1 2.496 Mus musculus aldehyde dehydrogenase family 3, subfamily A1 (Aldh3a1), transcript

variant 1, mRNA [NM_007436]

A_55_P1999301 Cyp2e1 2.474 Mus musculus cytochrome P450, family 2, subfamily e, polypeptide 1 (Cyp2e1), mRNA

[NM_021282]

A_66_P138319 Acox2 2.309 Mus musculus acyl-Coenzyme A oxidase 2, branched chain (Acox2), transcript variant 1,

mRNA [NM_053115]

A_51_P493649 Sult1e1 2.201 Mus musculus sulfotransferase family 1E, member 1 (Sult1e1), mRNA [NM_023135]

A_30_P01027986 2.116 lincRNA:chr8:19678750-20038750 reverse strand

A_55_P2124712 Ces2 2.063 Mus musculus carboxylesterase 2 (Ces2), mRNA [NM_145603]

A_51_P324814 Krt18 2.021 Mus musculus keratin 18 (Krt18), mRNA [NM_010664]

A_55_P2126448 1810032O08Rik 2.011 Mus musculus RIKEN cDNA 1810032O08 gene (1810032O08Rik), transcript variant 3,

non-coding RNA [NR_027821]

A_30_P01026441 2.007 lincRNA:chr14:77077374-77086910 forward strand

A_66_P107256 Kcna10 1.976 Mus musculus potassium voltage-gated channel, shaker-related subfamily, member 10

(Kcna10), mRNA [NM_001081140]

A_55_P2000833 E2f8 1.935 Mus musculus E2F transcription factor 8 (E2f8), mRNA [NM_001013368]

A_30_P01022068 1.914 lincRNA:chr10:126818382-126856582 reverse strand

A_51_P240253 Rrad 1.886 Mus musculus Ras-related associated with diabetes (Rrad), mRNA [NM_019662]

A_51_P173285 Nkx2-5 1.874 Mus musculus NK2 transcription factor related, locus 5 (Drosophila) (Nkx2-5), mRNA

[NM_008700]

A_51_P479352 Klhdc9 1.853 Mus musculus kelch domain containing 9 (Klhdc9), mRNA [NM_001033039]

A_30_P01020222 1.829 lincRNA:chr4:62367014-62386800 forward strand

A_51_P205390 1700040L02Rik 1.822 Mus musculus RIKEN cDNA 1700040L02 gene (1700040L02Rik), mRNA [NM_028491]

A_30_P01020559 1.816 lincRNA:chr2:151992806-152025456 reverse strand

A_66_P102528 Tspan5 1.815 Mus musculus tetraspanin 5 (Tspan5), mRNA [NM_019571]

A_55_P2057189 LOC100044832 1.785 PREDICTED: Mus musculus similar to RIKEN cDNA 1810030J14 gene (LOC100044832),

mRNA [XM_001472830]

A_55_P1994633 Gm711 1.781 Mus musculus predicted gene 711 (Gm711), mRNA [NM_198628]

A_55_P2033376 1810041L15Rik 1.780 Mus musculus RIKEN cDNA 1810041L15 gene (1810041L15Rik), mRNA

[NM_001163145]

A_51_P102122 Myog 1.764 Mus musculus myogenin (Myog), mRNA [NM_031189]

A_55_P2107065 Mcm8 1.735 Mus musculus minichromosome maintenance deficient 8 (S. cerevisiae) (Mcm8), mRNA

[NM_025676]

A_30_P01025529 1.735 lincRNA:chr14:78936896-78937160 reverse strand

A_30_P01026377 1.724 lincRNA:chr18:61781410-61788685 reverse strand

A_51_P351166 Myod1 1.706 Mus musculus myogenic differentiation 1 (Myod1), mRNA [NM_010866]

A_51_P472829 Aif1l 1.706 Mus musculus allograft inflammatory factor 1-like (Aif1l), mRNA [NM_145144]

A_55_P2168118 4933406C10Rik 1.674 PREDICTED: Mus musculus RIKEN cDNA 4933406C10 gene (4933406C10Rik), mRNA

[XM_001480777]

A_51_P382849 Emb 1.652 Mus musculus embigin (Emb), mRNA [NM_010330]

A_52_P139438 Fam150b 1.652 Mus musculus family with sequence similarity 150, member B (Fam150b), mRNA

[NM_001159743]

A_55_P2095064 LOC100045797 1.649 PREDICTED: Mus musculus hypothetical protein LOC100045797 (LOC100045797),

mRNA [XM_001474950]

A_51_P134030 Oas1e 1.643 Mus musculus 2'-5' oligoadenylate synthetase 1E (Oas1e), mRNA [NM_145210]

A_55_P2404554 2310050B05Rik 1.634 Mus musculus adult male epididymis cDNA, RIKEN full-length enriched library,

clone:9230023N21 product:hypothetical protein, full insert sequence. [AK136697]

A_55_P2125684 Camkk2 1.619 Mus musculus calcium/calmodulin-dependent protein kinase kinase 2, beta (Camkk2),

mRNA [NM_145358]

A_55_P2105944 LOC100044660 1.617 PREDICTED: Mus musculus similar to olfactory receptor (MOR275-3) pseudogene

(LOC100044660), mRNA [XM_001472787]

A_55_P1955188 Spata21 1.604 Mus musculus spermatogenesis associated 21 (Spata21), mRNA [NM_177867]

A_52_P496403 1700001O22Rik 1.601 Mus musculus RIKEN cDNA 1700001O22 gene (1700001O22Rik), transcript variant 1,

mRNA [NM_198000]

A_55_P2164988 Scrt1 1.587 Mus musculus scratch homolog 1, zinc finger protein (Drosophila) (Scrt1), mRNA

[NM_130893]

A_52_P320279 Inca1 1.579 Mus musculus inhibitor of CDK, cyclin A1 interacting protein 1 (Inca1), mRNA

[NM_213729]

A_51_P261059 Cpne2 1.567 Mus musculus copine II (Cpne2), mRNA [NM_153507]

A_55_P2156404 Ccdc136 1.561 Mus musculus coiled-coil domain containing 136 (Ccdc136), mRNA [NM_145574]

A_55_P1953402 Mlana 1.561 Mus musculus melan-A (Mlana), mRNA [NM_029993]

A_30_P01023982 1.558 lincRNA:chr6:90745692-90777977 forward strand

A_30_P01025950 1.553 lincRNA:chr12:16838729-16839360 forward strand

A_55_P2053057 Ppp2r1b 1.551 Mus musculus protein phosphatase 2 (formerly 2A), regulatory subunit A (PR 65), beta

isoform (Ppp2r1b), transcript variant 2, mRNA [NM_028614]

A_55_P2010567 Gm7564 1.550 PREDICTED: Mus musculus predicted gene, EG665273 (EG665273), mRNA

[XM_975768]

A_51_P114005 Gstm7 1.545 Mus musculus glutathione S-transferase, mu 7 (Gstm7), mRNA [NM_026672]

A_55_P2160543 Tcp10a 1.544 Mus musculus t-complex protein 10a (Tcp10a), mRNA [NM_009340]

A_55_P1994020 Casc4 1.541 Mus musculus cancer susceptibility candidate 4 (Casc4), transcript variant 1, mRNA

[NM_177054]

A_30_P01024389 1.535 lincRNA:chr9:30805020-30810645 reverse strand

A_55_P1997581 1.532

A_30_P01021191 1.521 lincRNA:chr1:36095790-36096193 reverse strand

A_66_P102903 Fez1 1.518 Mus musculus fasciculation and elongation protein zeta 1 (zygin I) (Fez1), mRNA

[NM_183171]

A_55_P2000034 Cryba4 1.515 Mus musculus crystallin, beta A4 (Cryba4), mRNA [NM_021351]

A_55_P2184796 Pcdhb18 1.515 Mus musculus protocadherin beta 18 (Pcdhb18), mRNA [NM_053143]

A_55_P2025268 Pqlc3 1.512 Mus musculus PQ loop repeat containing (Pqlc3), transcript variant 2, mRNA

[NM_001161111]

A_30_P01032219 1.501 lincRNA:chr12:110002978-110003360 reverse strand

A_30_P01030152 1.493 lincRNA:chr2:77146893-77155643 forward strand

A_55_P1999923 6430598A04Rik 1.493 Mus musculus RIKEN cDNA 6430598A04 gene (6430598A04Rik), mRNA [NM_175521]

A_51_P486748 Opn1mw 1.484 Mus musculus opsin 1 (cone pigments), medium-wave-sensitive (color blindness, deutan)

(Opn1mw), mRNA [NM_008106]

A_55_P2060764 Gm13807 1.480 PREDICTED: Mus musculus hypothetical LOC639658 (LOC639658), mRNA

[XM_001002949]

A_30_P01027508 1.477 lincRNA:chr2:158169020-158191295 reverse strand

A_30_P01022471 1.473 lincRNA:chr11:97505439-97522274 forward strand

A_55_P2383248 4930422N03Rik 1.472 Mus musculus adult male testis cDNA, RIKEN full-length enriched library,

clone:4930422N03 product:unclassifiable, full insert sequence. [AK015184]

A_30_P01020003 1.465 lincRNA:chr14:77511189-77522852 forward strand

A_51_P196925 Cx3cl1 1.460 Mus musculus chemokine (C-X3-C motif) ligand 1 (Cx3cl1), mRNA [NM_009142]

A_55_P2126269 Nmb 1.455 Mus musculus neuromedin B (Nmb), mRNA [NM_026523]

A_30_P01027524 1.451 lincRNA:chrX:150159252-150181275 reverse strand

A_55_P1989608 Olfr10 1.451 Mus musculus olfactory receptor 10 (Olfr10), mRNA [NM_206822]

A_30_P01024363 1.449 lincRNA:chr14:21462770-21463270 forward strand

A_30_P01019807 1.448 lincRNA:chr17:32035750-32056000 reverse strand

A_55_P2016526 Lrrc49 1.444 Mus musculus leucine rich repeat containing 49 (Lrrc49), transcript variant 2, mRNA

[NM_145616]

A_30_P01025498 1.443 lincRNA:chr16:89961600-89971725 forward strand

A_30_P01022497 1.442 lincRNA:chr1:134237581-134248506 reverse strand

A_55_P2096942 Nrn1l 1.442 Mus musculus neuritin 1-like (Nrn1l), mRNA [NM_175024]

A_55_P2004777 Micall2 1.439 Mus musculus MICAL-like 2 (Micall2), mRNA [NM_174850]

A_66_P103818 Elk1 1.438 Mus musculus ELK1, member of ETS oncogene family (Elk1), mRNA [NM_007922]

A_30_P01021437 1.435 lincRNA:chr17:32035750-32056000 reverse strand

A_55_P2122605 Cbr2 1.434 Mus musculus carbonyl reductase 2 (Cbr2), mRNA [NM_007621]

A_30_P01030217 1.429 lincRNA:chr12:73860757-73862034 reverse strand

A_55_P1954693 Tac1 1.428 Mus musculus tachykinin 1 (Tac1), mRNA [NM_009311]

A_55_P2074843 Ccdc122 1.427 Mus musculus coiled-coil domain containing 122 (Ccdc122), mRNA [NM_175369]

A_51_P293862 Gnb3 1.426 Mus musculus guanine nucleotide binding protein (G protein), beta 3 (Gnb3), mRNA

[NM_013530]

A_51_P370510 BC022687 1.425 Mus musculus cDNA sequence BC022687 (BC022687), mRNA [NM_145450]

A_51_P359293 Ubxn2b 1.416 Mus musculus UBX domain protein 2B (Ubxn2b), mRNA [NM_026534]

A_55_P2122604 Cbr2 1.415 Mus musculus carbonyl reductase 2 (Cbr2), mRNA [NM_007621]

A_55_P1994550 Shank1 1.413 Mus musculus SH3/ankyrin domain gene 1 (Shank1), mRNA [NM_001034115]

A_30_P01032529 1.412 lincRNA:chr17:85422370-85423168 forward strand

A_51_P420415 Srd5a1 1.410 Mus musculus steroid 5 alpha-reductase 1 (Srd5a1), mRNA [NM_175283]

A_55_P2385148 LOC552913 1.409 Mus musculus 16 days neonate cerebellum cDNA, RIKEN full-length enriched library,

clone:9630027L18 product:unclassifiable, full insert sequence. [AK036020]

A_55_P2064741 Nmb 1.409 Mus musculus neuromedin B (Nmb), mRNA [NM_026523]

A_55_P1995195 Fosl2 1.408 Mus musculus fos-like antigen 2 (Fosl2), mRNA [NM_008037]

A_51_P440238 Ggt6 1.407 Mus musculus gamma-glutamyltransferase 6 (Ggt6), mRNA [NM_027819]

A_51_P452203 Vash2 1.405 Mus musculus vasohibin 2 (Vash2), transcript variant 1, mRNA [NM_144879]

A_52_P58283 Kcna5 1.402 Mus musculus potassium voltage-gated channel, shaker-related subfamily, member 5

(Kcna5), mRNA [NM_145983]

A_30_P01023840 1.402 lincRNA:chr18:35204950-35216650 reverse strand

A_55_P2101920 4931408A02Rik 1.401 Mus musculus RIKEN cDNA 4931408A02 gene (4931408A02Rik), mRNA [NM_027627]

A_55_P1990866 Mylk3 1.397 Mus musculus myosin light chain kinase 3 (Mylk3), mRNA [NM_175441]

A_55_P1980601 Oscp1 1.396 Mus musculus organic solute carrier partner 1 (Oscp1), mRNA [NM_172701]

A_65_P20641 Fads2 1.394 Fatty acid desaturase 2 (EC 1.14.19.-)(Delta(6) fatty acid desaturase)(Delta(6)

desaturase)(D6D) [Source:UniProtKB/Swiss-Prot;Acc:Q9Z0R9]

[ENSMUST00000025567]

A_55_P1965876 Opn1mw 1.393 Mus musculus opsin 1 (cone pigments), medium-wave-sensitive (color blindness, deutan)

(Opn1mw), mRNA [NM_008106]

A_55_P2174661 Tmsb15b1-Tmsb15b2 1.392 Mus musculus Tmsb15b1-Tmsb15b2 readthrough transcript (Tmsb15b1-Tmsb15b2),

mRNA [NM_207267]

A_30_P01019750 1.392 lincRNA:chr17:32044823-32045974 reverse strand

A_55_P2028491 Hmgn2 1.385 Mus musculus high mobility group nucleosomal binding domain 2 (Hmgn2), mRNA

[NM_016957]

A_55_P2107987 9330176C04Rik 1.383 Mus musculus RIKEN cDNA 9330176C04 gene (9330176C04Rik), mRNA [NM_175420]

A_52_P480266 2610110G12Rik 1.382 Mus musculus RIKEN cDNA 2610110G12 gene (2610110G12Rik), transcript variant 3,

mRNA [NM_028476]

A_30_P01031419 1.376 lincRNA:chr1:138570107-138632310 forward strand

A_51_P490817 Me2 1.376 Mus musculus malic enzyme 2, NAD(+)-dependent, mitochondrial (Me2), nuclear gene

encoding mitochondrial protein, mRNA [NM_145494]

A_55_P2044992 Gm14025 1.375 PREDICTED: Mus musculus similar to catenin, alpha 2 (LOC668894), mRNA

[XM_001004454]

A_30_P01019229 1.371 lincRNA:chr12:33604247-33638654 reverse strand

A_52_P251623 BC057022 1.370 Mus musculus cDNA sequence BC057022 (BC057022), mRNA [NM_001004180]

A_55_P2076106 1.368 UPF0594 protein C13orf38 homolog [Source:UniProtKB/Swiss-Prot;Acc:Q8BXX9]

[ENSMUST00000118963]

A_30_P01032750 1.365 lincRNA:chr5:92556322-92558910 forward strand

A_51_P232748 Plxnb3 1.362 Mus musculus plexin B3 (Plxnb3), mRNA [NM_019587]

A_30_P01022897 1.362 lincRNA:chr9:113650590-113670786 forward strand

A_55_P1974947 Tmem232 1.362 Mus musculus transmembrane protein 232 (Tmem232), mRNA [NM_001008973]

A_51_P161463 Ccdc103 1.360 Mus musculus coiled-coil domain containing 103 (Ccdc103), mRNA [NM_028492]

A_55_P2117243 Npm1 1.360 Mus musculus 2 days neonate thymus thymic cells cDNA, RIKEN full-length enriched

library, clone:E430031K14 product:nucleophosmin 1, full insert sequence. [AK088923]

A_30_P01028196 1.358 lincRNA:chr19:46422690-46423478 reverse strand

A_55_P2094963 Gm10105 1.357 PREDICTED: Mus musculus similar to high mobility group nucleosomal binding domain 2

(LOC100047164), mRNA [XM_001477902]

A_30_P01025488 1.356 lincRNA:chr11:22681800-22707775 forward strand

A_51_P479818 Lonrf3 1.352 Mus musculus LON peptidase N-terminal domain and ring finger 3 (Lonrf3), mRNA

[NM_028894]

A_51_P144894 9230104L09Rik 1.351 Mus musculus RIKEN cDNA 9230104L09 gene (9230104L09Rik), mRNA [NM_029960]

A_30_P01017543 1.351 lincRNA:chr17:85418489-85427364 forward strand

A_55_P2054331 LOC236260 1.350 PREDICTED: Mus musculus hypothetical protein LOC236260 (LOC236260), mRNA

[XM_982830]

A_30_P01026300 1.350 lincRNA:chr4:114631940-114632618 reverse strand

A_30_P01023109 1.350 lincRNA:chr17:13008796-13023201 forward strand

A_51_P272106 Cirbp 1.347 Mus musculus cold inducible RNA binding protein (Cirbp), mRNA [NM_007705]

A_55_P2026214 Gm5899 1.347 PREDICTED: Mus musculus predicted gene, EG545982 (EG545982), mRNA

[XM_620527]

A_52_P185343 1.343 Guanine nucleotide-binding protein subunit alpha-13 (G-protein subunit alpha-13)(G

alpha-13) [Source:UniProtKB/Swiss-Prot;Acc:P27601] [ENSMUST00000106702]

A_55_P2157966 Mtap1a 1.342 Mus musculus microtubule-associated protein 1 A (Mtap1a), mRNA [NM_032393]

A_55_P2109505 Gm6594 1.342 PREDICTED: Mus musculus predicted gene, EG625514 (EG625514), mRNA

[XM_890094]

A_66_P107476 A930035D04Rik 1.339 PREDICTED: Mus musculus RIKEN cDNA A930035D04 gene (A930035D04Rik), mRNA

[XM_887910]

A_55_P2184279 4930556A17Rik 1.339 PREDICTED: Mus musculus RIKEN cDNA 4930556A17 gene (4930556A17Rik), mRNA

[XM_979591]

A_51_P329928 Phlda3 1.339 Mus musculus pleckstrin homology-like domain, family A, member 3 (Phlda3), mRNA

[NM_013750]

A_55_P2165324 Acsl3 1.338 Mus musculus acyl-CoA synthetase long-chain family member 3 (Acsl3), transcript variant

1, mRNA [NM_028817]

A_30_P01020388 1.338 lincRNA:chrX:47890565-47915775 forward strand

A_51_P404992 Efhc2 1.337 Mus musculus EF-hand domain (C-terminal) containing 2 (Efhc2), mRNA [NM_028916]

A_55_P2039842 Frmpd1 1.335 Mus musculus FERM and PDZ domain containing 1 (Frmpd1), mRNA [NM_001081172]

A_55_P1992421 Hmgb3 1.333 Mus musculus high mobility group box 3 (Hmgb3), mRNA [NM_008253]

A_55_P2112355 Gm7125 1.332 PREDICTED: Mus musculus predicted gene, EG633752 (EG633752), mRNA

[XM_980525]

A_55_P2049602 Kat2a 1.331 Mus musculus K(lysine) acetyltransferase 2A (Kat2a), transcript variant 1, mRNA

[NM_020004]

A_55_P2002351 Lats2 1.329 Mus musculus large tumor suppressor 2 (Lats2), transcript variant B, mRNA [NM_153382]

A_55_P2013928 Dhrs13 1.329 Mus musculus dehydrogenase/reductase (SDR family) member 13 (Dhrs13), mRNA

[NM_183286]

A_55_P1990919 Sepx1 1.328 Mus musculus selenoprotein X 1 (Sepx1), mRNA [NM_013759]

A_51_P436817 Dos 1.327 Mus musculus downstream of Stk11 (Dos), mRNA [NM_015761]

A_55_P1966189 Il4ra 1.326 Mus musculus interleukin 4 receptor, alpha (Il4ra), mRNA [NM_001008700]

A_30_P01030322 1.326 lincRNA:chr17:85418489-85427364 forward strand

A_55_P2162136 Hmgn2 1.325 Mus musculus high mobility group nucleosomal binding domain 2 (Hmgn2), mRNA

[NM_016957]

A_52_P367760 Calml4 1.324 Mus musculus calmodulin-like 4 (Calml4), transcript variant 1, mRNA [NM_138304]

A_30_P01025759 1.322 lincRNA:chr2:80338640-80339980 reverse strand

A_52_P651948 1700025K23Rik 1.322 Mus musculus RIKEN cDNA 1700025K23 gene (1700025K23Rik), mRNA [NM_183254]

A_52_P214630 Sox9 1.321 Mus musculus SRY-box containing gene 9 (Sox9), mRNA [NM_011448]

A_52_P653937 Rpl21 1.321 Mus musculus ribosomal protein L21 (Rpl21), mRNA [NM_019647]

A_55_P2038767 Fam49a 1.319 Mus musculus family with sequence similarity 49, member A (Fam49a),

transcript variant 1, mRNA [NM_029758]

A_51_P317941 Perp 1.317 Mus musculus PERP, TP53 apoptosis effector (Perp), mRNA [NM_022032]

A_30_P01033245 1.316 lincRNA:chr4:123361002-123364781 reverse strand

A_55_P1984561 Cpne2 1.315 Mus musculus copine II (Cpne2), mRNA [NM_153507]

A_55_P1964752 Slc23a3 1.315 Mus musculus solute carrier family 23 (nucleobase transporters), member 3 (Slc23a3),

mRNA [NM_194333]

A_30_P01031169 1.315 lincRNA:chr12:109603526-109647403 reverse strand

A_30_P01020675 1.313 lincRNA:chr16:23534663-23535172 forward strand

A_52_P657123 Gm10270 1.311 PREDICTED: Mus musculus similar to put. HMG-17 protein (LOC100040129), mRNA

[XM_001474128]

A_55_P2094964 Gm10105 1.311 PREDICTED: Mus musculus similar to high mobility group nucleosomal binding domain 2

(LOC100047164), mRNA [XM_001477902]

A_52_P256817 Zik1 1.309 Mus musculus zinc finger protein interacting with K protein 1 (Zik1), mRNA [NM_009577]

A_30_P01023817 1.309 lincRNA:chr17:22045002-22046886 reverse strand

A_55_P2117525 Rbm3 1.305 Mus musculus RNA binding motif protein 3 (Rbm3), transcript variant 3, mRNA

[NM_001166410]

A_55_P2071132 Tnfrsf23 1.304 Mus musculus tumor necrosis factor receptor superfamily, member 23 (Tnfrsf23), mRNA

[NM_024290]

A_55_P2135291 Gm7276 1.302 Mus musculus predicted gene 7276 (Gm7276), mRNA [NM_001039252]

A_52_P436318 Rps25 1.300 Mus musculus ribosomal protein S25 (Rps25), mRNA [NM_024266]

A_30_P01030915 1.299 lincRNA:chrX:47890565-47915775 forward strand

A_55_P2046288 Ccdc103 1.299 Mus musculus coiled-coil domain containing 103 (Ccdc103), mRNA [NM_028492]

A_30_P01031494 1.297 lincRNA:chr6:52046805-52072744 reverse strand

A_55_P2027022 Prdm1 1.297 Mus musculus PR domain containing 1, with ZNF domain (Prdm1), mRNA [NM_007548]

A_30_P01030779 1.291 lincRNA:chr18:84757475-84757969 reverse strand

A_51_P396752 Arl2bp 1.291 Mus musculus ADP-ribosylation factor-like 2 binding protein (Arl2bp), transcript variant 1,

mRNA [NM_024191]

A_30_P01024819 1.288 lincRNA:chr7:77481448-77495548 reverse strand

A_55_P2084512 LOC100048116 1.288 PREDICTED: Mus musculus similar to Jmy-pending protein (LOC100048116), mRNA

[XM_001479554]

A_30_P01031615 1.283 lincRNA:chr6:117829576-117834247 forward strand

A_52_P335064 Mustn1 1.282 Mus musculus musculoskeletal, embryonic nuclear protein 1 (Mustn1), mRNA

[NM_181390]

A_55_P2000158 Pik3r3 1.282 Mus musculus phosphatidylinositol 3 kinase, regulatory subunit, polypeptide 3 (p55)

(Pik3r3), mRNA [NM_181585]

A_55_P2168296 Rnu7 1.280 Mus musculus U7 small nuclear RNA (Rnu7), small nuclear RNA [NR_024201]

A_55_P2077628 1.279 L-xylulose reductase (XR)(EC 1.1.1.10)(Dicarbonyl/L-xylulose reductase)

[Source:UniProtKB/Swiss-Prot;Acc:Q91X52] [ENSMUST00000106148]

A_66_P100840 1.278

A_52_P141136 Fggy 1.278 Mus musculus FGGY carbohydrate kinase domain containing (Fggy), transcript variant 1,

mRNA [NM_001113412]

A_51_P133582 Cbx4 1.277 Mus musculus chromobox homolog 4 (Drosophila Pc class) (Cbx4), mRNA [NM_007625]

A_51_P181319 Dcxr 1.277 Mus musculus dicarbonyl L-xylulose reductase (Dcxr), mRNA [NM_026428]

A_55_P1988899 Mblac2 1.275 Mus musculus metallo-beta-lactamase domain containing 2 (Mblac2), mRNA

[NM_028372]

A_51_P143682 Zfp318 1.274 Mus musculus zinc finger protein 318 (Zfp318), transcript variant 2, mRNA [NM_021346]

A_55_P2165504 1.274

A_30_P01032630 1.273 lincRNA:chr15:5969454-5994540 reverse strand

A_55_P2120866 LOC100044171 1.273 PREDICTED: Mus musculus hypothetical protein LOC100044171 (LOC100044171),

mRNA [XM_001471569]

A_51_P262340 Rbm3 1.273 Mus musculus RNA binding motif protein 3 (Rbm3), transcript variant 2, mRNA

[NM_001166409]

A_30_P01021007 1.272 lincRNA:chr6:31001656-31002219 reverse strand

A_51_P375667 Tcea2 1.271 Mus musculus transcription elongation factor A (SII), 2 (Tcea2), mRNA [NM_009326]

A_55_P1956862 Egfr 1.270 Mus musculus epidermal growth factor receptor (Egfr), transcript variant 2, mRNA

[NM_007912]

A_52_P411331 Vac14 1.269 Mus musculus Vac14 homolog (S. cerevisiae) (Vac14), mRNA [NM_146216]

A_52_P153189 Arl2bp 1.269 Mus musculus ADP-ribosylation factor-like 2 binding protein (Arl2bp), transcript variant 2,

mRNA [NM_024269]

A_55_P2026753 Zmat1 1.266 Mus musculus zinc finger, matrin type 1 (Zmat1), mRNA [NM_175446]

A_55_P2143494 Doc2b 1.266 Mus musculus double C2, beta (Doc2b), mRNA [NM_007873]

A_55_P2206491 1810062O18Rik 1.266 Mus musculus 10 day old male pancreas cDNA, RIKEN full-length enriched library,

clone:1810062O18 product:hypothetical protein, full insert sequence. [AK007938]

A_30_P01018163 1.266 lincRNA:chrX:47890565-47915775 forward strand

A_52_P319774 Kcnip4 1.265 Mus musculus Kv channel interacting protein 4 (Kcnip4), mRNA [NM_030265]

A_55_P2038514 LOC100044759 1.264 PREDICTED: Mus musculus hypothetical protein LOC100044759 (LOC100044759),

mRNA [XM_001472992]

A_51_P181400 Efcab2 1.264 Mus musculus EF-hand calcium binding domain 2 (Efcab2), mRNA [NM_026626]

A_52_P173703 Myrip 1.261 Mus musculus myosin VIIA and Rab interacting protein (Myrip), mRNA [NM_144557]

A_52_P413646 Bmp6 1.261 Mus musculus bone morphogenetic protein 6 (Bmp6), mRNA [NM_007556]

A_65_P19442 Tatdn1 1.260 Mus musculus TatD DNase domain containing 1 (Tatdn1), mRNA [NM_175151]

A_55_P2028496 Hmgn2l6 1.260 PREDICTED: Mus musculus similar to high mobility group nucleosomal binding

domain 2, transcript variant 1 (LOC633596), mRNA [XM_001472085]

A_55_P2052240 Crlf1 1.259 Mus musculus cytokine receptor-like factor 1 (Crlf1), mRNA [NM_018827]

A_55_P2149144 4921517L17Rik 1.259 Mus musculus RIKEN cDNA 4921517L17 gene (4921517L17Rik), mRNA [NM_027585]

A_55_P2071799 Slbp 1.258 Mus musculus stem-loop binding protein (Slbp), mRNA [NM_009193]

A_55_P2092668 Ccbl2 1.258 Mus musculus cysteine conjugate-beta lyase 2 (Ccbl2), mRNA [NM_173763]

A_30_P01031572 1.258 lincRNA:chr8:74820025-74833350 forward strand

A_55_P2016105 Errfi1 1.257 Mus musculus ERBB receptor feedback inhibitor 1 (Errfi1), mRNA [NM_133753]

A_55_P2064351 Vipr1 1.256 Mus musculus vasoactive intestinal peptide receptor 1 (Vipr1), mRNA [NM_011703]

A_55_P1983853 Casp12 1.256 Mus musculus caspase 12 (Casp12), mRNA [NM_009808]

A_55_P2047614 Zfp809 1.254 Mus musculus zinc finger protein 809 (Zfp809), transcript variant 1, mRNA [NM_172763]

A_55_P1953593 Ece2 1.253 Mus musculus endothelin converting enzyme 2 (Ece2), transcript variant 3, mRNA

[NM_025462]

A_52_P123944 Mospd2 1.251 Mus musculus motile sperm domain containing 2 (Mospd2), mRNA [NM_029730]

A_55_P1990066 1.250

A_51_P131653 1700021C14Rik 1.250 Mus musculus RIKEN cDNA 1700021C14 gene (1700021C14Rik), mRNA [NM_029801]

A_52_P70854 Tob2 1.249 Mus musculus transducer of ERBB2, 2 (Tob2), mRNA [NM_020507]

A_52_P84901 Slc44a1 1.248 Mus musculus solute carrier family 44, member 1 (Slc44a1), transcript variant 1, mRNA

[NM_133891]

A_51_P132718 2310010J17Rik 1.248 PREDICTED: Mus musculus RIKEN cDNA 2310010J17 gene (2310010J17Rik), mRNA

[XM_896611]

A_55_P2320668 1700013G23Rik 1.247 AGENCOURT_10723978 NIH_MGC_169 Mus musculus cDNA clone IMAGE:

6772189 5', mRNA sequence [CA464421]

A_55_P1963364 Fam55c 1.247 Mus musculus family with sequence similarity 55, member C (Fam55c), mRNA

[NM_001134494]

A_30_P01029905 1.247 lincRNA:chr12:74135916-74142780 reverse strand

A_30_P01026316 1.243 lincRNA:chr8:74473445-74496948 forward strand

A_51_P337918 Aldh4a1 1.243 Mus musculus aldehyde dehydrogenase 4 family, member A1 (Aldh4a1), nuclear gene

encoding mitochondrial protein, mRNA [NM_175438]

A_55_P2028942 Ncam1 1.243 Mus musculus neural cell adhesion molecule 1 (Ncam1), transcript variant 2, mRNA

[NM_010875]

A_30_P01025384 1.243 lincRNA:chr12:82495575-82516300 forward strand

A_55_P1970846 Pemt 1.243 Mus musculus phosphatidylethanolamine N-methyltransferase (Pemt), nuclear gene

encoding mitochondrial protein, mRNA [NM_008819]

A_55_P2353004 4930568K20Rik 1.241 Mus musculus adult male testis cDNA, RIKEN full-length enriched library,

clone:4930568K20 product:unclassifiable, full insert sequence. [AK016253]

A_30_P01017693 1.241 lincRNA:chr8:122807653-122823000 reverse strand

A_55_P2136258 Nsun6 1.240 Mus musculus NOL1/NOP2/Sun domain family member 6 (Nsun6), transcript variant 1,

mRNA [NM_001165941]

A_66_P124952 Hspe1 1.239 Mus musculus heat shock protein 1 (chaperonin 10) (Hspe1), mRNA [NM_008303]

A_51_P156857 2010002N04Rik 1.238 Mus musculus RIKEN cDNA 2010002N04 gene (2010002N04Rik), mRNA [NM_134133]

A_30_P01019369 1.238 lincRNA:chr1:138528250-138538250 forward strand

A_30_P01020959 1.235 lincRNA:chr12:82495575-82516300 reverse strand

A_55_P1958137 1.235 PREDICTED: Mus musculus hypothetical protein LOC100043157 (LOC100043157),

mRNA [XM_001479647]

A_51_P219061 Oxa1l 1.235 Mus musculus oxidase assembly 1-like (Oxa1l), mRNA [NM_026936]

A_30_P01020198 1.234 lincRNA:chr17:17628846-17641021 reverse strand

A_55_P2166743 1.233 Putative uncharacterized proteinMCG1039900, isoform CRA_b ;

[Source:UniProtKB/TrEMBL;Acc:Q8CF42] [ENSMUST00000077161]

A_66_P138956 LOC100045396 1.233 PREDICTED: Mus musculus hypothetical protein LOC100045396 (LOC100045396),

mRNA [XM_001473828]

A_30_P01019519 1.232 lincRNA:chr5:122048289-122068947 reverse strand

A_52_P663600 Pak1 1.232 Mus musculus p21 protein (Cdc42/Rac)-activated kinase 1 (Pak1), mRNA [NM_011035]

A_55_P2173331 LOC100044193 1.232 Mus musculus hypothetical protein LOC100044193 (LOC100044193), mRNA

[NM_001024731]

A_55_P1999102 Pi16 1.232 Mus musculus peptidase inhibitor 16 (Pi16), mRNA [NM_023734]

A_51_P393067 Ap4m1 1.230 Mus musculus adaptor-related protein complex AP-4, mu 1 (Ap4m1), mRNA [NM_021392]

A_55_P2140449 B3galt4 1.229 Mus musculus UDP-Gal:betaGlcNAc beta 1,3-galactosyltransferase, polypeptide 4

(B3galt4), mRNA [NM_019420]

A_52_P199928 1.229 Cellular nucleic acid-binding protein (CNBP)(Zinc finger protein 9)

[Source:UniProtKB/Swiss-Prot;Acc:P53996] [ENSMUST00000113617]

A_55_P2017939 Lamc3 1.229 Mus musculus laminin gamma 3 (Lamc3), mRNA [NM_011836]

A_55_P2121456 Fam43a 1.228 Mus musculus family with sequence similarity 43, member A (Fam43a), mRNA

[NM_177632]

A_52_P646755 1700029F09Rik 1.227 Mus musculus RIKEN cDNA 1700029F09 gene (1700029F09Rik), mRNA [NM_029368]

A_51_P456857 Ift122 1.227 Mus musculus intraflagellar transport 122 homolog (Chlamydomonas) (Ift122), transcript

variant 1, mRNA [NM_031177]

A_55_P2010116 Rab27b 1.226 Mus musculus RAB27b, member RAS oncogene family (Rab27b), transcript variant 1,

mRNA [NM_030554]

A_55_P2197338 2010012P19Rik 1.226 Mus musculus adult male small intestine cDNA, RIKEN full-length enriched library,

clone:2010012P19 product:unclassifiable, full insert sequence. [AK008218]

A_51_P212741 Scn2b 1.225 Mus musculus sodium channel, voltage-gated, type II, beta (Scn2b), mRNA

[NM_001014761]

A_55_P1994942 Rorc 1.225 Mus musculus RAR-related orphan receptor gamma (Rorc), mRNA [NM_011281]

A_51_P177491 Ctrl 1.225 Mus musculus chymotrypsin-like (Ctrl), mRNA [NM_023182]

A_51_P488092 Pde6d 1.225 Mus musculus phosphodiesterase 6D, cGMP-specific, rod, delta (Pde6d), mRNA

[NM_008801]

A_30_P01033516 1.222 lincRNA:chr11:51854060-51903278 forward strand

A_55_P2056654 Kif22 1.221 Mus musculus kinesin family member 22 (Kif22), mRNA [NM_145588]

A_52_P428745 Camk2d 1.219 Mus musculus calcium/calmodulin-dependent protein kinase II, delta (Camk2d), transcript

variant 1, mRNA [NM_001025439]

A_51_P334419 1.219 Muscle, skeletal receptor tyrosine protein kinase Precursor (EC 2.7.10.1)(Muscle-specific

kinase receptor)(MuSK) [Source:UniProtKB/Swiss-Prot;Acc:Q61006]

[ENSMUST00000081919]

A_55_P1979457 Prdx4 1.218 Mus musculus peroxiredoxin 4 (Prdx4), mRNA [NM_016764]

A_55_P2051293 Tbcd 1.218 Mus musculus tubulin-specific chaperone d (Tbcd), mRNA [NM_029878]

A_30_P01028853 1.218 lincRNA:chr18:75513062-75523889 reverse strand

A_52_P552550 Agtrap 1.218 Mus musculus angiotensin II, type I receptor-associated protein (Agtrap), mRNA

[NM_009642]

A_30_P01025050 1.217 lincRNA:chr13:56286339-56298089 forward strand

A_55_P2089358 Hspe1 1.217 Mus musculus heat shock protein 1 (chaperonin 10) (Hspe1), mRNA [NM_008303]

A_55_P2166745 1.217 Putative uncharacterized proteinMCG1039900, isoform CRA_b ;

[Source:UniProtKB/TrEMBL;Acc:Q8CF42] [ENSMUST00000077161]

A_51_P185846 Dnalc4 1.217 Mus musculus dynein, axonemal, light chain 4 (Dnalc4), mRNA [NM_017470]

A_55_P2080016 Gm10649 1.217 Mus musculus predicted gene 10649 (Gm10649), non-coding RNA [NR_028579]

A_51_P341647 Fancc 1.216 Mus musculus Fanconi anemia, complementation group C (Fancc), transcript variant 1,

mRNA [NM_007985]

A_55_P2048493 4931440P22Rik 1.216 Mus musculus RIKEN cDNA 4931440P22 gene (4931440P22Rik), non-coding RNA

[NR_027955]

A_51_P369516 Nudt16l1 1.215 Mus musculus nudix (nucleoside diphosphate linked moiety X)-type motif 16-like 1

(Nudt16l1), mRNA [NM_025839]

A_65_P04980 Ilf3 1.214 Mus musculus interleukin enhancer binding factor 3 (Ilf3), transcript variant 3, mRNA

[NM_001042708]

A_55_P2120308 Cacna1a 1.214 Mus musculus calcium channel, voltage-dependent, P/Q type, alpha 1A subunit (Cacna1a),

mRNA [NM_007578]

A_52_P517063 Immp2l 1.213 Mus musculus IMP2 inner mitochondrial membrane peptidase-like (S. cerevisiae)

(Immp2l), nuclear gene encoding mitochondrial protein, mRNA [NM_053122]

A_51_P375509 Spty2d1 1.213 Mus musculus SPT2, Suppressor of Ty, domain containing 1 (S. cerevisiae) (Spty2d1),

mRNA [NM_175318]

A_51_P384113 9330129D05Rik 1.213 Mus musculus RIKEN cDNA 9330129D05 gene (9330129D05Rik), mRNA [NM_178799]

A_51_P414706 Ddt 1.213 Mus musculus D-dopachrome tautomerase (Ddt), mRNA [NM_010027]

A_55_P2048794 Cd82 1.212 Mus musculus CD82 antigen (Cd82), transcript variant 1, mRNA [NM_007656]

A_30_P01018584 1.212 lincRNA:chr4:40720362-40729687 forward strand

A_55_P2146500 1.212 Coiled-coil domain-containing protein 107 Precursor

[Source:UniProtKB/Swiss-Prot;Acc:Q9DCC3] [ENSMUST00000107922]

A_52_P130727 Ldoc1l 1.212 Mus musculus leucine zipper, down-regulated in cancer 1-like (Ldoc1l), mRNA

[NM_177630]

A_52_P496870 2310011J03Rik 1.211 Mus musculus RIKEN cDNA 2310011J03 gene (2310011J03Rik), mRNA [NM_025521]

A_30_P01023130 1.211 lincRNA:chr15:32167516-32174417 reverse strand

A_66_P118849 2310034G01Rik 1.211 PREDICTED: Mus musculus RIKEN cDNA 2310034G01 gene (2310034G01Rik), mRNA

[XM_001478866]

A_55_P2074499 Kcp 1.210 Mus musculus kielin/chordin-like protein (Kcp), mRNA [NM_001029985]

A_30_P01032767 1.209 lincRNA:chr9:88575075-88611650 reverse strand

A_30_P01022423 1.209 lincRNA:chr13:98038568-98068079 reverse strand

A_55_P2148431 Slc4a3 1.209 Mus musculus solute carrier family 4 (anion exchanger), member 3 (Slc4a3), mRNA

[NM_009208]

A_52_P447284 Clic6 1.209 Mus musculus chloride intracellular channel 6 (Clic6), mRNA [NM_172469]

A_55_P2104617 Kctd5 1.208 Mus musculus potassium channel tetramerisation domain containing 5 (Kctd5), mRNA

[NM_027008]

A_55_P2068148 Pias1 1.208 Mus musculus protein inhibitor of activated STAT 1 (Pias1), mRNA [NM_019663]

A_55_P2093439 Mex3d 1.208 Mus musculus mex3 homolog D (C. elegans) (Mex3d), mRNA [NM_198615]

A_55_P1973683 Morn4 1.208 Mus musculus MORN repeat containing 4 (Morn4), mRNA [NM_198108]

A_55_P2394308 Fst 1.208 Mus musculus follistatin (Fst), mRNA [NM_008046]

A_51_P276063 Phyh 1.207 Mus musculus phytanoyl-CoA hydroxylase (Phyh), mRNA [NM_010726]

A_66_P121583 Gm7120 1.207 Mus musculus predicted gene 7120 (Gm7120), mRNA [NM_001039244]

A_30_P01020654 1.206 lincRNA:chr14:67585234-67596823 reverse strand

A_55_P2094906 LOC637477 1.206 PREDICTED: Mus musculus hypothetical LOC637477 (LOC637477), mRNA

[XM_001476893]

A_52_P317393 Gpr56 1.206 Mus musculus G protein-coupled receptor 56 (Gpr56), mRNA [NM_018882]

A_52_P345247 Tfb1m 1.203 Mus musculus transcription factor B1, mitochondrial (Tfb1m), nuclear gene encoding

mitochondrial protein, mRNA [NM_146074]

A_55_P2180805 Pex3 1.203 Mus musculus peroxisomal biogenesis factor 3 (Pex3), transcript variant 2, mRNA

[NM_001164195]

A_55_P2078173 1.203

A_55_P2008889 Tmsb15b2 1.202 Mus musculus thymosin beta 15b2 (Tmsb15b2), mRNA [NM_001080967]

A_52_P200610 C1qtnf9 1.202 Mus musculus C1q and tumor necrosis factor related protein 9 (C1qtnf9), mRNA

[NM_183175]

A_55_P2068731 Ccl27a 1.201 Mus musculus chemokine (C-C motif) ligand 27A (Ccl27a), transcript variant 4, mRNA

[NM_001164045]

A_30_P01021640 1.200 lincRNA:chr6:86776350-86799379 reverse strand

Decreased

A_30_P01022557 3.711 lincRNA:chr13:24615451-24615973 forward strand

A_30_P01028966 3.163 lincRNA:chr6:99472981-99484210 reverse strand

A_51_P303424 Itgax 2.926 Mus musculus integrin alpha X (Itgax), mRNA [NM_021334]

A_55_P1979904 Mup9 2.887 Mus musculus major urinary protein 9 (Mup9), mRNA [NM_001126319]

A_51_P256827 S100a8 2.817 Mus musculus S100 calcium binding protein A8 (calgranulin A) (S100a8), mRNA [NM_013650]

A_55_P1952618 Ear2 2.758 Mus musculus eosinophil-associated, ribonuclease A family, member 2 (Ear2), mRNA

[NM_007895]

A_55_P2144914 LOC100047516 2.584 PREDICTED: Mus musculus similar to casein kinase 1, gamma 3, transcript variant 1

(LOC100047516), mRNA [XM_001478504]

A_30_P01022531 2.521 lincRNA:chr17:22076711-22099431 reverse strand

A_55_P1954985 Foxn3 2.471 PREDICTED: Mus musculus hypothetical protein LOC100039892 (LOC100039892),

mRNA [XM_001473733]

A_55_P2124233 Vsig8 2.256 Mus musculus V-set and immunoglobulin domain containing 8 (Vsig8), transcript variant 1,

mRNA [NM_177723]

A_30_P01021865 2.247 lincRNA:chr2:157361470-157379813 reverse strand

A_30_P01019058 2.207 lincRNA:chr2:156654675-156665430 forward strand

A_55_P2090787 2.185 Mitochondrial tumor suppressor 1 homolog (Angiotensin-II type 2 receptor-interacting

protein)(AT2 receptor-binding protein)(Coiled-coiled tumor suppressor gene 1 protein)

[Source:UniProtKB/Swiss-Prot;Acc:Q5HZI1] [ENSMUST00000070824]

A_55_P2020806 V1rc15 2.113 Mus musculus vomeronasal 1 receptor, C15 (V1rc15), mRNA [NM_134170]

A_55_P2040951 Actc1 2.109 Mus musculus actin, alpha, cardiac muscle 1 (Actc1), mRNA [NM_009608]

A_55_P1987585 LOC100045012 2.050 PREDICTED: Mus musculus hypothetical protein LOC100045012 (LOC100045012),

mRNA [XM_001473504]

A_55_P2395911 D2Ertd295e 2.040 Mus musculus 16 days neonate cerebellum cDNA, RIKEN full-length enriched library,

clone:9630008E13 product:unclassifiable, full insert sequence. [AK035822]

A_30_P01024650 2.020 lincRNA:chr1:85304494-85305390 reverse strand

A_55_P2279997 4930549C15Rik 1.991 Mus musculus adult male testis cDNA, RIKEN full-length enriched library,

clone:4930549C15 product:unclassifiable, full insert sequence. [AK016075]

A_52_P75777 LOC676300 1.969 PREDICTED: Mus musculus similar to leucine-rich repeat-containing G protein-coupled

receptor 6 (LOC676300), mRNA [XM_988868]

A_55_P1975330 1.969 Putative uncharacterized protein [Source:UniProtKB/TrEMBL;Acc:Q3ULS7]

[ENSMUST00000099221]

A_30_P01033454 1.966 lincRNA:chr1:85300475-85331475 reverse strand

A_51_P245989 Ccr2 1.952 Mus musculus chemokine (C-C motif) receptor 2 (Ccr2), mRNA [NM_009915]

A_55_P2184013 C130026I21Rik 1.902 Mus musculus RIKEN cDNA C130026I21 gene (C130026I21Rik), transcript variant 1,

mRNA [NM_175219]

A_55_P2165199 Cxcr6 1.893 Mus musculus chemokine (C-X-C motif) receptor 6 (Cxcr6), mRNA [NM_030712]

A_55_P2010093 Mup4 1.893 Mus musculus major urinary protein 4 (Mup4), mRNA [NM_008648]

A_65_P04847 Vgll3 1.890 Mus musculus vestigial like 3 (Drosophila) (Vgll3), mRNA [NM_028572]

A_55_P1981167 C130026I21Rik 1.882 Mus musculus RIKEN cDNA C130026I21 gene (C130026I21Rik), transcript variant 1,

mRNA [NM_175219]

A_55_P2186080 Gm12680 1.875 PREDICTED: Mus musculus hypothetical protein A930009N24 (A930009N24), mRNA

[XM_987909]

A_55_P2186220 LOC100039029 1.868 PREDICTED: Mus musculus similar to HSR (LOC100039029), mRNA [XM_001472058]

A_30_P01029551 1.862 lincRNA:chr15:61984389-62102500 forward strand

A_30_P01026003 1.861 lincRNA:chr11:96175764-96176029 forward strand

A_55_P2187067 1.855 Immunoglobulin Kappa light chain V gene segment

[Source:IMGT/GENE-DB;Acc:IGKV1-117] [ENSMUST00000103317]

A_30_P01031082 1.843 lincRNA:chr12:52886460-52888921 reverse strand

A_30_P01018756 1.842 lincRNA:chr14:65433420-65434061 reverse strand

A_55_P2237117 LOC552878 1.819 Mus musculus 16 days embryo head cDNA, RIKEN full-length enriched library,

clone:C130023C23 product:unclassifiable, full insert sequence. [AK047948]

A_55_P2087292 1.813 T-cell receptor beta V gene segment [Source:IMGT/GENE-DB;Acc:TRBV12-2]

[ENSMUST00000103269]

A_52_P538927 Zfp385b 1.804 Mus musculus zinc finger protein 385B (Zfp385b), transcript variant 1, mRNA

[NM_178723]

A_30_P01030738 1.803 lincRNA:chr1:85304494-85305390 reverse strand

A_55_P2197134 A930018M24Rik 1.797 Mus musculus adult male diencephalon cDNA, RIKEN full-length enriched library,

clone:9330167E06 product:unclassifiable, full insert sequence. [AK034241]

A_66_P128445 1.769 BEN domain-containing protein 7 [Source:UniProtKB/Swiss-Prot;Acc:Q8BSV3]

[ENSMUST00000115022]

A_55_P2294295 Tanc2 1.760 Mus musculus tetratricopeptide repeat, ankyrin repeat and coiled-coil containing 2 (Tanc2),

mRNA [NM_181071]

A_30_P01019505 1.752 lincRNA:chr16:34524589-34573439 reverse strand

A_30_P01020016 1.748 lincRNA:chr13:54399275-54445825 reverse strand

A_55_P2179290 Gm3622 1.742 PREDICTED: Mus musculus similar to tau tubulin kinase 1 (LOC100042007), mRNA

[XM_001477370]

A_30_P01021613 1.736 lincRNA:chr12:110944403-110988678 forward strand

A_55_P2394968 C530030K21Rik 1.736 Mus musculus 0 day neonate lung cDNA, RIKEN full-length enriched library,

clone:E030042J04 product:unclassifiable, full insert sequence. [AK087292]

A_30_P01027216 1.735 lincRNA:chr6:83367433-83370986 reverse strand

A_55_P2060238 Itgbl1 1.729 Mus musculus integrin, beta-like 1 (Itgbl1), mRNA [NM_145467]

A_55_P2019267 Gm4473 1.727 PREDICTED: Mus musculus hypothetical protein LOC100044460 (LOC100044460),

mRNA [XM_001472465]

A_30_P01019436 1.721 lincRNA:chr4:129497990-129515290 reverse strand

A_51_P277088 Igfals 1.718 Mus musculus insulin-like growth factor binding protein, acid labile subunit (Igfals),

mRNA [NM_008340]

A_30_P01024522 1.712 lincRNA:chr2:143991240-143991597 forward strand

A_55_P2054409 Pira2 1.696 Mus musculus paired-Ig-like receptor A2 (Pira2), mRNA [NM_011089]

A_55_P2017710 Igsf9 1.688 Mus musculus immunoglobulin superfamily, member 9 (Igsf9), transcript variant 1, mRNA

[NM_033608]

A_55_P2059680 Gm11938 1.676 Mus musculus predicted gene 11938 (Gm11938), mRNA [NM_001127354]

A_30_P01027817 1.675 lincRNA:chr7:66926500-66952100 forward strand

A_55_P2052416 Iqsec1 1.675 Mus musculus IQ motif and Sec7 domain 1 (Iqsec1), transcript variant 1, mRNA

[NM_001134383]

A_55_P2178653 LOC675548 1.673 PREDICTED: Mus musculus similar to putative retrovirus-related gag protein

(LOC675548), mRNA [XM_983026]

A_30_P01029737 1.658 lincRNA:chr2:60038543-60046318 reverse strand

A_30_P01024461 1.656 lincRNA:chr14:65205076-65219432 reverse strand

A_30_P01024874 1.656 lincRNA:chr8:119090566-119097735 forward strand

A_55_P2085142 Spp1 1.653 Mus musculus secreted phosphoprotein 1 (Spp1), mRNA [NM_009263]

A_30_P01032568 1.649 lincRNA:chr4:40840725-40860875 forward strand

A_55_P2435613 Xpo7 1.648 Mus musculus exportin 7 (Xpo7), mRNA [NM_023045]

A_55_P2012171 Spata6 1.648 Mus musculus spermatogenesis associated 6 (Spata6), mRNA [NM_026470]

A_30_P01029874 1.642 lincRNA:chr16:59557120-59602070 forward strand

A_55_P2373987 A730009E18Rik 1.642 Mus musculus 16 days embryo head cDNA, RIKEN full-length enriched library,

clone:C130001E07 product:unclassifiable, full insert sequence. [AK047818]

A_30_P01033651 1.642 lincRNA:chr4:101010948-101032148 forward strand

A_55_P2121772 Ano8 1.636 Mus musculus anoctamin 8 (Ano8), mRNA [NM_001164679]

A_30_P01022116 1.633 lincRNA:chr16:4525147-4542122 forward strand

A_55_P2007964 Cx3cr1 1.626 Mus musculus chemokine (C-X3-C) receptor 1 (Cx3cr1), mRNA [NM_009987]

A_55_P2330600 4930554C24Rik 1.625 Mus musculus adult male testis cDNA, RIKEN full-length enriched library,

clone:4930554C24 product:unclassifiable, full insert sequence. [AK016115]

A_55_P2041678 Ttc38 1.619 Mus musculus tetratricopeptide repeat domain 38 (Ttc38), mRNA [NM_001033337]

A_55_P2255944 9130002K18Rik 1.618 Mus musculus adult male cecum cDNA, RIKEN full-length enriched library,

clone:9130002K18 product:hypothetical protein, full insert sequence. [AK018586]

A_66_P112113 1.616 Protein kinase C theta type (EC 2.7.11.13)(nPKC-theta)

[Source:UniProtKB/Swiss-Prot;Acc:Q02111] [ENSMUST00000102970]

A_55_P2046145 1.612 Mus musculus adult male bone cDNA, RIKEN full-length enriched library,

clone:9830001O09 product:hypothetical protein, full insert sequence. [AK137448]

A_30_P01022855 1.609 lincRNA:chr11:96175764-96176029 forward strand

A_55_P2199777 5830443J22Rik 1.608 Mus musculus adult male thymus cDNA, RIKEN full-length enriched library,

clone:5830443J22 product:unclassifiable, full insert sequence. [AK017987]

A_30_P01028666 1.605 lincRNA:chr3:30100736-30101280 reverse strand

A_30_P01030860 1.601 lincRNA:chr13:62734450-62785325 reverse strand

A_30_P01026896 1.596 lincRNA:chr9:114412295-114418384 forward strand

A_30_P01021795 1.593 lincRNA:chr2:155269145-155303251 forward strand

A_55_P1992834 Socs2 1.591 Mus musculus suppressor of cytokine signaling 2 (Socs2), transcript variant 1, mRNA

[NM_007706]

A_30_P01030921 1.591 lincRNA:chr7:4827340-4863315 reverse strand

A_30_P01027573 1.590 lincRNA:chr1:85036677-85038530 reverse strand

A_55_P2029905 1.589 Receptor-type tyrosine-protein phosphatase S Precursor (R-PTP-S)(EC

3.1.3.48)(Receptor-type tyrosine-protein phosphatase sigma)(R-PTP-sigma)(PTPNU-3)

[Source:UniProtKB/Swiss-Prot;Acc:B0V2N1] [ENSMUST00000097302]

A_51_P107362 Socs2 1.586 Mus musculus suppressor of cytokine signaling 2 (Socs2), transcript variant 1, mRNA

[NM_007706]

A_51_P242493 Ankrd60 1.586 PREDICTED: Mus musculus RIKEN cDNA 1700030G11 gene (1700030G11Rik), mRNA

[XM_130735]

A_55_P2349752 8030494B02Rik 1.586 Mus musculus 15 days embryo male testis cDNA, RIKEN full-length enriched library,

clone:8030494B02 product:hypothetical protein, full insert sequence. [AK136342]

A_52_P336585 Als2 1.586 Mus musculus amyotrophic lateral sclerosis 2 (juvenile) homolog (human) (Als2), transcript

variant 1, mRNA [NM_028717]

A_30_P01024442 1.582 lincRNA:chr6:47687025-47728525 reverse strand

A_52_P235861 Peg3 1.576 Mus musculus paternally expressed 3 (Peg3), mRNA [NM_008817]

A_55_P1999162 LOC100041081 1.576 PREDICTED: Mus musculus similar to RIKEN cDNA 5830484A20 gene

(LOC100041081), mRNA [XM_001475751]

A_30_P01027571 1.574 lincRNA:chr4:136856064-136875614 reverse strand

A_55_P2435563 Etl4 1.567 Mus musculus enhancer trap locus 4 (Etl4), mRNA [NM_001081006]

A_55_P2403874 D230014I24Rik 1.567 Mus musculus 12 days embryo eyeball cDNA, RIKEN full-length enriched library,

clone:D230014I24 product:retinoblastoma binding protein 2, full insert sequence.

[AK051876]

A_55_P2025687 Muc4 1.567 Mus musculus mucin 4 (Muc4), mRNA [NM_080457]

A_55_P2359436 A930026B05Rik 1.567 Mus musculus adult retina cDNA, RIKEN full-length enriched library, clone:A930026B05

product:unclassifiable, full insert sequence. [AK020890]

A_55_P2059527 Gon4l 1.566 Mus musculus gon-4-like (C.elegans) (Gon4l), mRNA [NM_027389]

A_30_P01028152 1.562 lincRNA:chr5:111538420-111540142 forward strand

A_52_P207303 Bmp3 1.560 Mus musculus bone morphogenetic protein 3 (Bmp3), mRNA [NM_173404]

A_55_P2201454 2310079F09Rik 1.559 Mus musculus 12 days embryo eyeball cDNA, RIKEN full-length enriched library,

clone:D230018B15 product:unclassifiable, full insert sequence. [AK142062]

A_52_P602188 Sned1 1.557 Mus musculus sushi, nidogen and EGF-like domains 1 (Sned1), mRNA [NM_172463]

A_55_P2116165 Pfkfb3 1.555 Mus musculus 6-phosphofructo-2-kinase/fructose-2,6-biphosphatase 3 (Pfkfb3), mRNA

[NM_133232]

A_55_P2091393 1.545 Putative uncharacterized protein [Source:UniProtKB/TrEMBL;Acc:Q3USB9]

[ENSMUST00000072180]

A_55_P2158962 Dixdc1 1.544 Mus musculus DIX domain containing 1 (Dixdc1), mRNA [NM_178118]

A_30_P01027807 1.543 lincRNA:chr2:177256373-177257429 forward strand

A_51_P282557 C3ar1 1.542 Mus musculus complement component 3a receptor 1 (C3ar1), mRNA [NM_009779]

A_55_P1993925 1.541 Putative uncharacterized protein [Source:UniProtKB/TrEMBL;Acc:Q3U0W5]

[ENSMUST00000115260]

A_55_P1992838 Socs2 1.541 Mus musculus suppressor of cytokine signaling 2 (Socs2), transcript variant 1, mRNA

[NM_007706]

A_51_P369426 Zmynd17 1.541 Mus musculus zinc finger, MYND domain containing 17 (Zmynd17), mRNA

[NM_029104]

A_30_P01021999 1.540 lincRNA:chr16:34559623-34560462 reverse strand

A_55_P1964902 Gm3014 1.539 PREDICTED: Mus musculus similar to CG2839-PA (LOC100040872), mRNA

[XM_001475360]

A_55_P2450933 N4bp2l2 1.534 Mus musculus NEDD4 binding protein 2-like 2 (N4bp2l2), mRNA [NM_201369]

A_30_P01025934 1.534 lincRNA:chr12:21548850-21568550 reverse strand

A_55_P2099267 LOC100045917 1.532 PREDICTED: Mus musculus hypothetical protein LOC100045917 (LOC100045917),

mRNA [XM_001475176]

A_55_P2153762 LOC640020 1.519 PREDICTED: Mus musculus similar to intestinal mucin (LOC640020), mRNA

[XM_916728]

A_55_P2225470 A230092J17Rik 1.519 Mus musculus adult male hypothalamus cDNA, RIKEN full-length enriched library,

clone:A230092J17 product:unclassifiable, full insert sequence. [AK039069]

A_52_P520495 Vcam1 1.518 Mus musculus vascular cell adhesion molecule 1 (Vcam1), mRNA [NM_011693]

A_52_P362917 Pfkfb3 1.516 Mus musculus 6-phosphofructo-2-kinase/fructose-2,6-biphosphatase 3 (Pfkfb3), mRNA

[NM_133232]

A_55_P2106345 1.516 AT-rich interactive domain-containing protein 4B (ARID domain-containing protein

4B)(Histone deacetylase complex subunit SAP180)(180 kDa Sin3-associated

polypeptide)(Sin3-associated polypeptide p180)

[Source:UniProtKB/Swiss-Prot;Acc:A2CG63] [ENSMUST00000110533]

A_55_P2043362 Gm4021 1.515 PREDICTED: Mus musculus hypothetical protein LOC100042769 (LOC100042769),

mRNA [XM_001478906]

A_55_P2040838 Gm14548 1.506 Mus musculus predicted gene 14548 (Gm14548), mRNA [NM_001166672]

A_30_P01019267 1.503 lincRNA:chr4:136856064-136875614 reverse strand

A_30_P01019500 1.503 lincRNA:chr8:19682258-19700836 forward strand

A_55_P2345116 1700109K24Rik 1.499 Mus musculus 12 days embryo spinal ganglion cDNA, RIKEN full-length enriched library,

clone:D130035C10 product:hypothetical protein, full insert sequence. [AK051328]

A_30_P01025731 1.495 lincRNA:chrX:148732853-148761972 reverse strand

A_55_P2193424 6720420G18Rik 1.495 Mus musculus 12 days embryo male wolffian duct includes surrounding region cDNA,

RIKEN full-length enriched library, clone:6720420G18 product:unclassifiable, full insert

sequence. [AK020112]

A_30_P01024665 1.493 lincRNA:chr12:111829967-111830904 forward strand

A_30_P01018482 1.493 lincRNA:chr12:5303823-5379098 reverse strand

A_30_P01027162 1.490 lincRNA:chr12:52880692-52933444 reverse strand

A_55_P2045741 Fyb 1.488 Mus musculus FYN binding protein (Fyb), mRNA [NM_011815]

A_30_P01031309 1.487 lincRNA:chr2:48805604-48974396 forward strand

A_30_P01019384 1.485 lincRNA:chr13:28551902-28975446 reverse strand

A_30_P01022510 1.484 lincRNA:chr10:96103732-96103867 forward strand

A_30_P01026651 1.484 lincRNA:chr6:90848183-90861277 reverse strand

A_55_P1964896 Gm3014 1.484 PREDICTED: Mus musculus similar to CG2839-PA (LOC100040872), mRNA

[XM_001475360]

A_30_P01026609 1.482 lincRNA:chr18:36449530-36456800 reverse strand

A_66_P112305 Myo1f 1.481 Mus musculus myosin IF (Myo1f), mRNA [NM_053214]

A_30_P01019726 1.480 lincRNA:chr16:64820905-64822069 reverse strand

A_55_P2258832 5230400M03Rik 1.478 Mus musculus adult male xiphoid cartilage cDNA, RIKEN full-length enriched library,

clone:5230400M03 product:unclassifiable, full insert sequence. [AK030345]

A_55_P2202524 9330162012Rik 1.477 Mus musculus adult male diencephalon cDNA, RIKEN full-length enriched library,

clone:9330162O12 product:unclassifiable, full insert sequence. [AK034200]

A_52_P419632 Srcap 1.477 PREDICTED: Mus musculus similar to Snf2-related CBP activator protein

(LOC100043597), mRNA [XM_001480403]

A_52_P867058 Kcnc1 1.476 Mus musculus potassium voltage gated channel, Shaw-related subfamily, member 1

(Kcnc1), transcript variant A, mRNA [NM_001112739]

A_30_P01019184 1.475 lincRNA:chr1:85036677-85038530 reverse strand

A_55_P1995108 5430427O19Rik 1.471 Mus musculus RIKEN cDNA 5430427O19 gene (5430427O19Rik), mRNA

[NM_001163539]

A_55_P2200029 Dleu2 1.471 Mus musculus adult male cortex cDNA, RIKEN full-length enriched library,

clone:7730401J12 product:inferred: deleted in lymphocytic leukemia, 2, full insert

sequence. [AK033078]

A_30_P01022708 1.470 lincRNA:chr9:67480847-67481797 reverse strand

A_55_P2042016 LOC100048058 1.469 PREDICTED: Mus musculus similar to Solute carrier family 29 (nucleoside transporters),

member 4 (LOC100048058), mRNA [XM_001479435]

A_30_P01032372 1.466 lincRNA:chr18:69923410-69965360 forward strand

A_30_P01019295 1.464 lincRNA:chr16:64823448-64824632 reverse strand

A_55_P2189893 A630073K07Rik 1.462 Mus musculus 3 days neonate thymus cDNA, RIKEN full-length enriched library,

clone:A630073K07 product:unclassifiable, full insert sequence. [AK042233]

A_30_P01026562 1.462 lincRNA:chr3:41143423-41359623 forward strand

A_55_P2018330 Gm13298 1.460 Mus musculus predicted gene 13298 (Gm13298), mRNA [NM_001085530]

A_55_P2015892 1.460 WW domain-containing oxidoreductase (EC 1.1.1.-)

[Source:UniProtKB/Swiss-Prot;Acc:Q91WL8] [ENSMUST00000109106]

A_55_P2214487 E330013P08Rik 1.459 Mus musculus 7 days neonate cerebellum cDNA, RIKEN full-length enriched library,

clone:A730077H03 product:unclassifiable, full insert sequence. [AK043262]

A_55_P1966216 Gm14700 1.458 PREDICTED: Mus musculus hypothetical protein LOC100040255 (LOC100040255),

mRNA [XM_001474363]

A_55_P2175440 LOC100045610 1.457 PREDICTED: Mus musculus hypothetical protein LOC100045610 (LOC100045610),

mRNA [XM_001474598]

A_55_P2252184 C76336 1.457 M15E-1429 MOUSE EMBRYONIC DAY 15.5 EYE Mus musculus cDNA 5', mRNA

sequence [CB840983]

A_30_P01020492 1.456 lincRNA:chr11:95644899-95658549 reverse strand

A_55_P2115364 Gm4429 1.456 PREDICTED: Mus musculus similar to C11G6.3 (LOC100043430), mRNA

[XM_001480151]

A_30_P01023264 1.455 lincRNA:chr7:134375931-134376644 reverse strand

A_55_P2302383 3300002A11Rik 1.453 Mus musculus 11 days embryo whole body cDNA, RIKEN full-length enriched library,

clone:2700016B18 product:unclassifiable, full insert sequence. [AK012244]

A_30_P01031231 1.448 lincRNA:chr16:11008534-11013759 forward strand

A_30_P01019691 1.448 lincRNA:chr12:3199327-3200827 forward strand

A_51_P290931 1.445 protein tyrosine phosphatase, receptor type, B [Source:RefSeq peptide;Acc:NP_084204]

[ENSMUST00000020363]

A_55_P2371984 5930436O19Rik 1.444 Mus musculus 13 days embryo forelimb cDNA, RIKEN full-length enriched library,

clone:5930436O19 product:unclassifiable, full insert sequence. [AK020044]

A_30_P01033351 1.444 lincRNA:chr8:18587198-18592948 reverse strand

A_55_P2171493 BC030867 1.442 Mus musculus cDNA sequence BC030867 (BC030867), mRNA [NM_153544]

A_55_P2384593 Gpc6 1.442 Mus musculus glypican 6 (Gpc6), transcript variant 1, mRNA [NM_001079844]

A_55_P2055607 Enpp1 1.441 Mus musculus ectonucleotide pyrophosphatase/phosphodiesterase 1 (Enpp1), mRNA

[NM_008813]

A_30_P01021750 1.441 lincRNA:chr16:32388843-32395264 reverse strand

A_55_P2079560 Pira3 1.441 Mus musculus paired-Ig-like receptor A3 (Pira3), transcript variant 1, mRNA [NM_011090]

A_55_P2046328 Gm6225 1.440 PREDICTED: Mus musculus predicted gene, EG621440, transcript variant 1 (EG621440),

mRNA [XM_973616]

A_55_P1973415 Csprs 1.439 Mus musculus component of Sp100-rs (Csprs), mRNA [NM_033616]

A_55_P2072005 1.436 Dipeptidyl-peptidase 1 Precursor (EC 3.4.14.1)(Dipeptidyl-peptidase

I)(DPP-I)(DPPI)(Cathepsin C)(Cathepsin J)(Dipeptidyl transferase) [Contains

Dipeptidyl-peptidase 1 exclusion domain chain(Dipeptidyl-peptidase I exclusion domain

chain);Dipeptidyl-peptidase 1 heavy chain(Dipeptidyl-peptidase I heavy

chain);Dipeptidyl-peptidase 1 light chain(Dipeptidyl-peptidase I light chain)]

[Source:UniProtKB/Swiss-Prot;Acc:P97821] [ENSMUST00000098313]

A_30_P01021410 1.435 lincRNA:chr5:22801058-22939737 reverse strand

A_55_P2060922 Unc5a 1.430 Mus musculus unc-5 homolog A (C. elegans) (Unc5a), mRNA [NM_153131]

A_51_P258372 Igsf6 1.430 Mus musculus immunoglobulin superfamily, member 6 (Igsf6), mRNA [NM_030691]

A_52_P444785 1.429 NEDD4-binding protein 2-like 2 (Phosphonoformate immuno-associated protein 5

homolog) [Source:UniProtKB/Swiss-Prot;Acc:Q8JZS6] [ENSMUST00000016274]

A_30_P01032304 1.428 lincRNA:chr3:30032959-30079659 reverse strand

A_51_P275053 1700094C09Rik 1.427 Mus musculus RIKEN cDNA 1700094C09 gene (1700094C09Rik), mRNA [NM_024279]

A_55_P2045855 6330416L07Rik 1.427 Mus musculus RIKEN cDNA 6330416L07 gene (6330416L07Rik), mRNA [NM_176962]

A_30_P01019105 1.427 lincRNA:chr8:48480224-48480698 reverse strand

A_55_P2130501 LOC100044727 1.426 PREDICTED: Mus musculus hypothetical protein LOC100044727 (LOC100044727),

mRNA [XM_001472813]

A_55_P2183110 Fmr1 1.425 Mus musculus fragile X mental retardation syndrome 1 homolog (Fmr1), mRNA

[NM_008031]

A_55_P2324921 0610039H22Rik 1.422 Mus musculus 12 days embryo eyeball cDNA, RIKEN full-length enriched library,

clone:D230019E14 product:unclassifiable, full insert sequence. [AK084302]

A_51_P205820 Klf11 1.422 Mus musculus Kruppel-like factor 11 (Klf11), mRNA [NM_178357]

A_55_P2380806 C77713 1.420 Mus musculus adult male urinary bladder cDNA, RIKEN full-length enriched library,

clone:9530027F24 product:unclassifiable, full insert sequence. [AK137174]

A_55_P2012999 Ank3 1.419 Mus musculus ankyrin 3, epithelial (Ank3), transcript variant 1, mRNA [NM_170728]

A_51_P507832 Pik3cg 1.419 Mus musculus phosphoinositide-3-kinase, catalytic, gamma polypeptide (Pik3cg), transcript

variant 1, mRNA [NM_020272]

A_55_P2398270 5033423O07Rik 1.418 Mus musculus 16 days neonate thymus cDNA, RIKEN full-length enriched library,

clone:A130052I20 product:unclassifiable, full insert sequence. [AK037820]

A_55_P2030045 Barx2 1.418 Mus musculus BarH-like homeobox 2 (Barx2), mRNA [NM_013800]

A_51_P248335 1.416 Zinc finger protein 618 [Source:UniProtKB/Swiss-Prot;Acc:Q80YY7]

[ENSMUST00000030043]

A_30_P01033460 1.415 lincRNA:chr1:6199946-6200603 reverse strand

A_55_P2383877 2810040C05Rik 1.413 Mus musculus 10, 11 days embryo whole body cDNA, RIKEN full-length enriched library,

clone:2810040C05 product:unclassifiable, full insert sequence. [AK012892]

A_51_P313503 Olfr577 1.411 Mus musculus olfactory receptor 577 (Olfr577), mRNA [NM_147109]

A_55_P2097533 Ankrd6 1.409 Mus musculus ankyrin repeat domain 6 (Ankrd6), transcript variant 1, mRNA

[NM_001012450]

A_55_P2127473 1.405 hypothetical protein LOC214642 [Source:RefSeq peptide;Acc:NP_001074820]

[ENSMUST00000115382]

A_52_P551856 Rnft1 1.404 Mus musculus ring finger protein, transmembrane 1 (Rnft1), mRNA [NM_029788]

A_30_P01029720 1.403 lincRNA:chr19:5842306-5845476 reverse strand

A_30_P01018790 1.402 lincRNA:chr4:155585650-155608700 forward strand

A_30_P01018542 1.399 lincRNA:chr1:171402829-171403289 reverse strand

A_55_P2178137 3-Sep 1.397 Mus musculus septin 3 (Sept3), mRNA [NM_011889]

A_55_P1969007 Asb18 1.395 Mus musculus ankyrin repeat and SOCS box-containing 18 (Asb18), mRNA [NM_139152]

A_30_P01023999 1.394 lincRNA:chr8:71891610-71924216 forward strand

A_55_P2076481 LOC100047656 1.394 PREDICTED: Mus musculus similar to novel KRAB box and zinc finger, C2H2 type

domain containing protein (LOC100047656), mRNA [XM_001478606]

A_55_P2097518 Zdhhc22 1.393 Mus musculus zinc finger, DHHC-type containing 22 (Zdhhc22), mRNA [NM_001080943]

A_55_P1994504 1.393 Spectrin beta 5 Fragment [Source:UniProtKB/TrEMBL;Acc:A2AP60]

[ENSMUST00000099515]

A_55_P1997106 Gyltl1b 1.392 Mus musculus glycosyltransferase-like 1B (Gyltl1b), transcript variant 1, mRNA

[NM_172670]

A_55_P2103026 Sema3d 1.392 Mus musculus sema domain, immunoglobulin domain (Ig), short basic domain, secreted,

(semaphorin) 3D (Sema3d), mRNA [NM_028882]

A_55_P2404733 LOC552909 1.392 Mus musculus 16 days embryo head cDNA, RIKEN full-length enriched library,

clone:C130020I06 product:unclassifiable, full insert sequence. [AK081485]

A_55_P2117380 Asb4 1.391 Mus musculus ankyrin repeat and SOCS box-containing 4 (Asb4), mRNA [NM_023048]

A_55_P2192819 BB157357 1.389 CJ239155 RIKEN full-length enriched mouse cDNA library, C57BL [CJ239155]

A_55_P2078680 Tpm1 1.388 Mus musculus tropomyosin 1, alpha (Tpm1), transcript variant 5, mRNA [NM_001164251]

A_52_P267824 2210023G05Rik 1.387 Mus musculus RIKEN cDNA 2210023G05 gene (2210023G05Rik), mRNA [NM_197999]

A_52_P337326 Kcnc3 1.387 Mus musculus potassium voltage gated channel, Shaw-related subfamily, member 3

(Kcnc3), mRNA [NM_008422]

A_55_P2411747 AI596198 1.386 AGENCOURT_15763788 NIH_MGC_204 Mus musculus cDNA clone IMAGE:30526659

5', mRNA sequence [CF617181]

A_55_P2362601 D4Ertd681e 1.386 Mus musculus 11 days embryo gonad cDNA, RIKEN full-length enriched library,

clone:7030402E17 product:DNA segment, Chr 4, ERATO Doi 681, expressed, full insert

sequence. [AK078562]

A_30_P01032845 1.385 lincRNA:chr16:93121505-93142255 reverse strand

A_30_P01023279 1.385 lincRNA:chr8:26343277-26352902 reverse strand

A_55_P2204396 6030458E02Rik 1.384 Mus musculus 13 days embryo male testis cDNA, RIKEN full-length enriched library,

clone:6030458E02 product:unclassifiable, full insert sequence. [AK020075]

A_55_P2021560 1.384 Putative uncharacterized protein [Source:UniProtKB/TrEMBL;Acc:Q8C3M0]

[ENSMUST00000059529]

A_55_P2047130 1.383 Putative uncharacterized protein Fragment [Source:UniProtKB/TrEMBL;Acc:Q3UTG7]

[ENSMUST00000097865]

A_55_P2333126 E330018D03Rik 1.381 Mus musculus 2 days neonate thymus thymic cells cDNA, RIKEN full-length enriched

library, clone:E430019C04 product:unclassifiable, full insert sequence. [AK088506]

A_30_P01030815 1.381 lincRNA:chr18:61809278-61825191 reverse strand

A_55_P2060386 Col15a1 1.380 Mus musculus collagen, type XV, alpha 1 (Col15a1), mRNA [NM_009928]

A_30_P01030216 1.380 lincRNA:chr14:62294849-62301165 reverse strand

A_55_P1983016 1.379 PREDICTED: Mus musculus hypothetical protein LOC100046491 (LOC100046491),

mRNA [XM_001476704]

A_30_P01030418 1.379 lincRNA:chr8:70623390-70645212 reverse strand

A_30_P01032825 1.377 lincRNA:chr13:27124478-27124684 forward strand

A_52_P30312 Ccr9 1.376 Mus musculus chemokine (C-C motif) receptor 9 (Ccr9), transcript variant 2, mRNA

[NM_009913]

A_30_P01027630 1.376 lincRNA:chr13:12792150-12869825 forward strand

A_55_P2176792 Sh3gl3 1.376 Mus musculus SH3-domain GRB2-like 3 (Sh3gl3), mRNA [NM_017400]

A_55_P2172742 1.375 Mitofusin-2 (EC 3.6.5.-)(Transmembrane GTPase MFN2)(Hypertension-related protein

1)(Mitochondrial assembly regulatory factor)(HSG protein)

[Source:UniProtKB/Swiss-Prot;Acc:Q80U63] [ENSMUST00000105714]

A_30_P01020135 1.374 lincRNA:chr19:5842306-5845476 reverse strand

A_30_P01025039 1.374 lincRNA:chr17:12941161-12944859 forward strand

A_55_P2230968 D930050J11 1.374 Mus musculus 15 days embryo head cDNA, RIKEN full-length enriched library,

clone:D930050J11 product:unclassifiable, full insert sequence. [AK086771]

A_30_P01017919 1.373 lincRNA:chr4:82156098-82196298 reverse strand

A_30_P01023578 1.372 lincRNA:chr14:62294849-62301165 reverse strand

A_55_P2044193 1.372 Vacuolar protein sorting-associated protein 29 (EC 3.1.3.3)(Vesicle protein sorting 29)

[Source:UniProtKB/Swiss-Prot;Acc:Q9QZ88] [ENSMUST00000117868]

A_30_P01018715 1.370 lincRNA:chr8:48480224-48480698 reverse strand

A_55_P2046910 Gm2405 1.369 PREDICTED: Mus musculus similar to dynein, axonemal, heavy chain 9 (LOC100039754),

mRNA [XM_001473440]

A_30_P01025429 1.368 lincRNA:chr1:108664564-108665243 reverse strand

A_52_P135873 Gm2744 1.366 PREDICTED: Mus musculus hypothetical protein LOC100040381 (LOC100040381),

mRNA [XM_001475453]

A_30_P01027707 1.366 lincRNA:chr13:99469411-99469882 forward strand

A_52_P493091 Fmr1 1.365 Mus musculus fragile X mental retardation syndrome 1 homolog (Fmr1), mRNA

[NM_008031]

A_30_P01020133 1.362 lincRNA:chr13:12792150-12869825 reverse strand

A_30_P01032902 1.362 lincRNA:chr17:15111725-15139800 forward strand

A_55_P2079064 Ppnr 1.361 PREDICTED: Mus musculus hypothetical protein LOC100044198 (LOC100044198),

mRNA [XM_001471675]

A_55_P2045632 Myo15b 1.361 PREDICTED: Mus musculus myosin XVB (Myo15b), mRNA [XM_981684]

A_55_P2306893 4931406E20Rik 1.361 Mus musculus 0 day neonate cerebellum cDNA, RIKEN full-length enriched library,

clone:C230022K22 product:Musashi homolog 2 (Drosophila), full insert sequence.

[AK163891]

A_52_P409961 Ccnt2 1.360 Mus musculus cyclin T2 (Ccnt2), mRNA [NM_028399]

A_55_P2317341 D13Ertd787e 1.360 H3036C01-5 NIA Mouse 15K cDNA Clone Set Mus musculus cDNA clone H3036C01 5',

mRNA sequence [BG079138]

A_55_P2166799 1.358

A_55_P2107682 1.357

A_30_P01024586 1.356 lincRNA:chr13:34693404-34717987 reverse strand

A_52_P491554 Zfp759 1.356 Mus musculus zinc finger protein 759 (Zfp759), mRNA [NM_172392]

A_30_P01033240 1.355 lincRNA:chr8:47813487-47824869 forward strand

A_55_P2113723 1.355 Peroxisomal trans-2-enoyl-CoA reductase (EC 1.3.1.38)

[Source:UniProtKB/Swiss-Prot;Acc:Q99MZ7] [ENSMUST00000097698]

A_55_P2001173 1.354 Putative uncharacterized protein [Source:UniProtKB/TrEMBL;Acc:Q3UW10]

[ENSMUST00000100177]

A_55_P2035038 Gm2437 1.353 PREDICTED: Mus musculus hypothetical protein LOC100039816 (LOC100039816),

mRNA [XM_001473590]

A_52_P478187 1.352 hypothetical protein LOC244425 [Source:RefSeq peptide;Acc:NP_848850]

[ENSMUST00000036104]

A_30_P01026948 1.351 lincRNA:chr2:155269145-155303251 reverse strand

A_30_P01030446 1.350 lincRNA:chr12:110909258-110913909 forward strand

A_30_P01021631 1.350 lincRNA:chr19:5842306-5845476 reverse strand

A_55_P2062084 LOC100044403 1.350 PREDICTED: Mus musculus similar to gag polyprotein (LOC100044403), mRNA

[XM_001472077]

A_55_P1996711 Olfr1443 1.349 Mus musculus olfactory receptor 1443 (Olfr1443), mRNA [NM_146698]

A_55_P2072522 Pcdhb16 1.348 Mus musculus protocadherin beta 16 (Pcdhb16), mRNA [NM_053141]

A_55_P2156370 BB014433 1.347 Mus musculus expressed sequence BB014433 (BB014433), mRNA [NM_001007591]

A_30_P01019146 1.346 lincRNA:chr2:128211990-128215223 reverse strand

A_52_P164286 Dnm3 1.345 Mus musculus dynamin 3 (Dnm3), transcript variant 1, mRNA [NM_001038619]

A_55_P1991475 Sesn1 1.345 Mus musculus sestrin 1 (Sesn1), transcript variant 1, mRNA [NM_001162908]

A_30_P01026028 1.345 lincRNA:chr5:23195569-23218551 reverse strand

A_55_P1982891 Klk9 1.344 Mus musculus kallikrein related-peptidase 9 (Klk9), mRNA [NM_028660]

A_55_P2302413 BC002189 1.343 Mus musculus cDNA sequence BC002189, mRNA (cDNA clone MGC:7386

IMAGE:3487872), complete cds. [BC002189]

A_52_P538673 Fgf1 1.342 Mus musculus fibroblast growth factor 1 (Fgf1), mRNA [NM_010197]

A_55_P1995354 Btnl2 1.341 Mus musculus butyrophilin-like 2 (Btnl2), mRNA [NM_079835]

A_55_P2100375 Col11a1 1.340 Mus musculus collagen, type XI, alpha 1 (Col11a1), mRNA [NM_007729]

A_55_P2009857 1.340

A_51_P322871 Sh3bp4 1.339 Mus musculus SH3-domain binding protein 4 (Sh3bp4), mRNA [NM_133816]

A_55_P2063505 Gm4235 1.338 PREDICTED: Mus musculus hypothetical protein LOC100043109 (LOC100043109),

mRNA [XM_001479508]

A_30_P01019616 1.338 lincRNA:chr10:126831225-126832253 forward strand

A_30_P01019458 1.338 lincRNA:chr5:15033568-15033987 forward strand

A_55_P2263053 D17892 1.337 AGENCOURT_16688470 NIH_MGC_203 Mus musculus cDNA clone IMAGE:30791167

5', mRNA sequence [CK128530]

A_51_P446469 Dok2 1.336 Mus musculus docking protein 2 (Dok2), mRNA [NM_010071]

A_55_P2243768 D930030O05Rik 1.335 Mus musculus 9 days embryo whole body cDNA, RIKEN full-length enriched library,

clone:D030028K24 product:unclassifiable, full insert sequence. [AK083489]

A_55_P2157448 Abr 1.335 Mus musculus active BCR-related gene (Abr), transcript variant 1, mRNA [NM_198018]

A_30_P01026556 1.333 lincRNA:chr16:3872469-3877048 forward strand

A_55_P2105517 1.331 PREDICTED: Mus musculus similar to CG2861-PB (LOC100045557), mRNA

[XM_001473956]

A_51_P460954 Ccl6 1.331 Mus musculus chemokine (C-C motif) ligand 6 (Ccl6), mRNA [NM_009139]

A_55_P2326507 A630057N01Rik 1.330 Mus musculus adult male diencephalon cDNA, RIKEN full-length enriched library,

clone:9330195C05 product:weakly similar to X-LINKED RETINOPATHY PROTEIN

(FRAGMENT) [Homo sapiens], full insert sequence. [AK079103]

A_30_P01022310 1.330 lincRNA:chr5:35975265-35982561 forward strand

A_52_P678117 Xpr1 1.330 Mus musculus xenotropic and polytropic retrovirus receptor 1 (Xpr1), mRNA

[NM_011273]

A_51_P162676 Armc7 1.330 Mus musculus armadillo repeat containing 7 (Armc7), mRNA [NM_177778]

A_51_P116665 LOC636687 1.328 PREDICTED: Mus musculus similar to 6820431F20Rik protein (LOC636687), mRNA

[XM_912173]

A_55_P1989495 1.328 Transmembrane protein 164 [Source:UniProtKB/Swiss-Prot;Acc:Q6PHN7]

[ENSMUST00000112897]

A_55_P2211793 6720474J12Rik 1.327 Mus musculus 12 days embryo male wolffian duct includes surrounding region cDNA,

RIKEN full-length enriched library, clone:6720474J12 product:unclassifiable, full insert

sequence. [AK032932]

A_30_P01025238 1.326 lincRNA:chr2:73224880-73255276 forward strand

A_55_P2200618 LOC552902 1.326 Mus musculus 16 days embryo head cDNA, RIKEN full-length enriched library,

clone:C130023P07 product:unclassifiable, full insert sequence. [AK047962]

A_55_P2072041 LOC100038980 1.325 PREDICTED: Mus musculus hypothetical protein LOC100038980 (LOC100038980),

mRNA [XM_001472087]

ERCC-00138_246 1.325 Unknown

A_55_P2046728 Gm7792 1.324 PREDICTED: Mus musculus predicted gene, EG665802, transcript variant 6 (EG665802),

mRNA [XM_981891]

A_30_P01029370 1.324 lincRNA:chr11:95627874-95640824 forward strand

A_55_P2238079 A830054O07Rik 1.323 Mus musculus 13 days embryo forelimb cDNA, RIKEN full-length enriched library,

clone:5930437J24 product:unclassifiable, full insert sequence. [AK031253]

A_66_P134171 1.322 Immunoglobulin Kappa light chain V gene segment

[Source:IMGT/GENE-DB;Acc:IGKV8-24] [ENSMUST00000103384]

A_55_P2419961 Ptpn4 1.322 Mus musculus protein tyrosine phosphatase, non-receptor type 4 (Ptpn4), mRNA

[NM_019933]

A_30_P01024755 1.322 lincRNA:chr10:66760153-66881675 forward strand

A_30_P01024606 1.321 lincRNA:chr5:35975265-35982561 forward strand

A_30_P01017675 1.320 lincRNA:chr17:12965849-12968519 forward strand

A_55_P1992164 E130102H24Rik 1.320 PREDICTED: Mus musculus RIKEN cDNA E130102H24 gene (E130102H24Rik), mRNA

[XM_001472210]

A_55_P2177551 Gm4981 1.320 Mus musculus predicted gene 4981 (Gm4981), mRNA [NM_001034869]

A_30_P01024986 1.320 lincRNA:chr8:106619342-106624851 reverse strand

A_55_P2341890 4921511C10Rik 1.319 Mus musculus 16 days neonate thymus cDNA, RIKEN full-length enriched library,

clone:A130040N09 product:unclassifiable, full insert sequence. [AK137931]

A_55_P2212458 LOC548102 1.319 Mus musculus 10 days neonate cerebellum cDNA, RIKEN full-length enriched library,

clone:B930001M17 product:unclassifiable, full insert sequence. [AK046899]

A_55_P2128270 Phf21a 1.318 Mus musculus PHD finger protein 21A (Phf21a), transcript variant 1, mRNA [NM_138755]

A_55_P2078650 1.318 Methyltransferase-like protein 5 (EC 2.1.1.-) [Source:UniProtKB/Swiss-Prot;Acc:Q8K1A0]

[ENSMUST00000112258]

A_55_P1969742 Gm9684 1.318 PREDICTED: Mus musculus hypothetical LOC676575 (LOC676575), mRNA

[XM_001480427]

A_55_P2058467 Gm4415 1.317 PREDICTED: Mus musculus hypothetical protein LOC100043402 (LOC100043402),

mRNA [XM_001479915]

A_30_P01028783 1.316 lincRNA:chr16:43623581-43642715 forward strand

A_51_P289679 Npm2 1.313 Mus musculus nucleophosmin/nucleoplasmin 2 (Npm2), mRNA [NM_181345]

A_55_P2372877 A330062J17Rik 1.313 Mus musculus adult male spinal cord cDNA, RIKEN full-length enriched library,

clone:A330062J17 product:unclassifiable, full insert sequence. [AK039561]

A_30_P01027691 1.313 lincRNA:chr12:109993800-109994116 reverse strand

A_51_P463428 Pik3ip1 1.311 Mus musculus phosphoinositide-3-kinase interacting protein 1 (Pik3ip1), mRNA

[NM_178149]

A_55_P2030135 1.311 RNA-binding protein 39 (RNA-binding motif protein 39)(RNA-binding region-containing

protein 2)(Coactivator of activating protein 1 and estrogen receptors)(Coactivator of AP-1

and ERs)(Transcription coactivator CAPER) [Source:UniProtKB/Swiss-Prot;Acc:Q8VH51]

[ENSMUST00000109584]

A_66_P110687 Taf1a 1.310 Mus musculus TATA box binding protein (Tbp)-associated factor, RNA polymerase I, A

(Taf1a), mRNA [NM_021466]

A_55_P2281624 2610034E01Rik 1.310 Mus musculus 13 days embryo heart cDNA, RIKEN full-length enriched library,

clone:D330027A05 product:unclassifiable, full insert sequence. [AK142273]

A_55_P2167930 1.309 Dystrophin [Source:UniProtKB/Swiss-Prot;Acc:P11531] [ENSMUST00000101404]

A_30_P01025647 1.306 lincRNA:chr2:174852887-174853961 forward strand

A_66_P134149 Snx30 1.305 Mus musculus sorting nexin family member 30 (Snx30), mRNA [NM_172468]

A_55_P2060091 1.305 Rab GTPase-binding effector protein 1 (Rabaptin-5)(Rabaptin-5alpha)

[Source:UniProtKB/Swiss-Prot;Acc:O35551] [ENSMUST00000108533]

A_51_P142107 Fzd7 1.305 Mus musculus frizzled homolog 7 (Drosophila) (Fzd7), mRNA [NM_008057]

A_55_P2034355 1.305 Eukaryotic translation initiation factor 4 gamma 3 (eIF-4-gamma 3)(eIF-4G 3)(eIF4G

3)(eIF-4-gamma II)(eIF4GII) [Source:UniProtKB/Swiss-Prot;Acc:Q80XI3]

[ENSMUST00000105829]

A_55_P2110537 Slfn9 1.304 Mus musculus schlafen 9 (Slfn9), mRNA [NM_172796]

A_30_P01031712 1.303 lincRNA:chr3:137736327-137752677 forward strand

A_30_P01029059 1.303 lincRNA:chr9:88333945-88342370 reverse strand

A_30_P01023574 1.303 lincRNA:chr16:64758224-64765700 reverse strand

A_55_P2141866 9630025I21Rik 1.303 Mus musculus RIKEN cDNA 9630025I21 gene (9630025I21Rik), mRNA

[NM_001076791]

A_55_P2143522 Fstl3 1.302 Mus musculus follistatin-like 3 (Fstl3), mRNA [NM_031380]

A_55_P2259500 D130012P04Rik 1.302 Mus musculus 12 days embryo spinal ganglion cDNA, RIKEN full-length enriched library,

clone:D130012P04 product:hypothetical protein, full insert sequence. [AK083809]

A_51_P240421 Pcdhb14 1.302 Mus musculus protocadherin beta 14 (Pcdhb14), mRNA [NM_053139]

A_55_P2176489 1.301 ATP-binding cassette sub-family D member 1 (Adrenoleukodystrophy protein)(ALDP)

[Source:UniProtKB/Swiss-Prot;Acc:P48410] [ENSMUST00000114461]

A_30_P01021593 1.301 lincRNA:chr5:35975265-35982561 forward strand

A_55_P2137466 Kcnu1 1.301 Mus musculus potassium channel, subfamily U, member 1 (Kcnu1), mRNA [NM_008432]

A_55_P2014315 Gm9581 1.299 PREDICTED: Mus musculus similar to CG6004-PB (LOC672884), mRNA

[XM_001480343]

A_30_P01022949 1.299 lincRNA:chr8:106619342-106624851 reverse strand

A_51_P129962 LOC100048649 1.299 PREDICTED: Mus musculus similar to VWF-cleaving protease Adamts-13

(LOC100048649), mRNA [XM_001480796]

A_30_P01019885 1.298 lincRNA:chr14:65433420-65434061 reverse strand

A_55_P2048358 Wfdc16 1.296 Mus musculus WAP four-disulfide core domain 16 (Wfdc16), mRNA [NM_001012723]

A_30_P01018898 1.295 lincRNA:chr15:10367307-10370076 reverse strand

A_30_P01031065 1.295 lincRNA:chr3:137734560-137740335 forward strand

A_55_P2064171 1.295

A_65_P01643 Chd2 1.295 Mus musculus chromodomain helicase DNA binding protein 2 (Chd2), mRNA

[NM_001081345]

A_30_P01022026 1.295 lincRNA:chr10:59382822-59383642 reverse strand

A_55_P2149812 1.294 splicing factor, arginine/serine-rich 11 isoform 1 [Source:RefSeq

peptide;Acc:NP_001087222] [ENSMUST00000098488]

A_55_P2202539 BC028789 1.293 Mus musculus cDNA sequence BC028789, mRNA (cDNA clone IMAGE:1263252), with

apparent retained intron. [BC028789]

A_55_P2024874 Lrp3 1.293 Mus musculus low density lipoprotein receptor-related protein 3 (Lrp3), mRNA

[NM_001024707]

A_30_P01029060 1.293 lincRNA:chr3:51890691-51891781 reverse strand

A_30_P01026880 1.292 lincRNA:chr15:68161768-68193518 reverse strand

A_55_P2120141 Gm4522 1.292 PREDICTED: Mus musculus similar to reproductive homeobox on X chromosome 8

(LOC100043565), mRNA [XM_001480410]

A_30_P01029782 1.292 lincRNA:chr5:88995039-89009064 reverse strand

A_66_P112065 Sertad2 1.291 Mus musculus 13 days embryo heart cDNA, RIKEN full-length enriched library,

clone:D330025G16 product:unclassifiable, full insert sequence. [AK142268]

A_55_P2064025 1.290 PREDICTED: Mus musculus similar to ORF1 (LOC100045623), mRNA [XM_001474990]

A_55_P2082841 Gm2658 1.290 PREDICTED: Mus musculus similar to Ubtf protein (LOC100040199), mRNA

[XM_001474255]

A_30_P01023802 1.288 lincRNA:chr5:23195569-23218551 reverse strand

A_55_P2019294 Gm8220 1.288 PREDICTED: Mus musculus hypothetical LOC676390 (LOC676390), mRNA

[XM_989725]

A_30_P01031474 1.288 lincRNA:chr15:85510625-85511265 forward strand

A_55_P1969221 Mrpl41 1.288 Mus musculus mitochondrial ribosomal protein L41 (Mrpl41), nuclear gene encoding

mitochondrial protein, mRNA [NM_001031808]

A_55_P2179493 1.288 Putative uncharacterized protein [Source:UniProtKB/TrEMBL;Acc:Q8C4H7]

[ENSMUST00000056274]

A_55_P2424627 4833427F10Rik 1.288 Mus musculus 0 day neonate head cDNA, RIKEN full-length enriched library,

clone:4833427F10 product:unclassifiable, full insert sequence. [AK014779]

A_51_P158018 Bend5 1.287 Mus musculus BEN domain containing 5 (Bend5), mRNA [NM_026279]

A_30_P01021541 1.287 lincRNA:chr15:61984389-62102500 reverse strand

A_30_P01028217 1.286 lincRNA:chr13:48620882-48643532 forward strand

A_55_P2255628 9430013L14Rik 1.286 Mus musculus 12 days embryo embryonic body between diaphragm region and neck

cDNA, RIKEN full-length enriched library, clone:9430013L14 product:unclassifiable, full

insert sequence. [AK020413]

A_55_P2108275 Gm4578 1.286 PREDICTED: Mus musculus similar to Ubtf protein (LOC100043666), mRNA

[XM_001480646]

A_52_P70787 Brdt 1.286 Mus musculus bromodomain, testis-specific (Brdt), transcript variant 1, mRNA

[NM_054054]

A_30_P01030794 1.285 lincRNA:chr8:72076364-72077503 reverse strand

A_55_P2006255 Txnip 1.285 Mus musculus thioredoxin interacting protein (Txnip), transcript variant 1, mRNA

[NM_001009935]

A_52_P533724 Ino80 1.285 Mus musculus INO80 homolog (S. cerevisiae) (Ino80), mRNA [NM_026574]

A_55_P2101944 Gm9444 1.285 PREDICTED: Mus musculus hypothetical LOC669185 (LOC669185), mRNA

[XM_001479912]

A_55_P2101666 Vamp5 1.284 Mus musculus vesicle-associated membrane protein 5 (Vamp5), transcript variant 1, mRNA

[NM_016872]

A_55_P2344463 LOC553089 1.284 Mus musculus 0 day neonate cerebellum cDNA, RIKEN full-length enriched library,

clone:C230091N07 product:unclassifiable, full insert sequence. [AK049016]

A_55_P2391674 4931407E12Rik 1.283 Mus musculus adult male testis cDNA, RIKEN full-length enriched library,

clone:4931407E12 product:unclassifiable, full insert sequence. [AK016440]

A_30_P01027849 1.280 lincRNA:chr8:41941084-41947630 reverse strand

A_52_P22331 1.280 A kinase anchor protein 10, mitochondrial Precursor (Protein kinase A-anchoring protein

10)(PRKA10)(Dual specificity A kinase-anchoring protein 2)(D-AKAP-2)

[Source:UniProtKB/Swiss-Prot;Acc:O88845] [ENSMUST00000108710]

A_55_P2081925 Zfp53 1.280 Mus musculus zinc finger protein 53 (Zfp53), mRNA [NM_013843]

A_55_P2332761 4930453H23Rik 1.278 Mus musculus adult male testis cDNA, RIKEN full-length enriched library,

clone:4930453H23 product:unclassifiable, full insert sequence. [AK015451]

A_55_P2281891 Bnc2 1.278 Mus musculus basonuclin 2 (Bnc2), mRNA [NM_172870]

A_52_P14526 Zyg11b 1.278 Mus musculus zyg-ll homolog B (C. elegans) (Zyg11b), mRNA [NM_001033634]

A_55_P1977303 1.277 Dual 3',5'-cyclic-AMP and -GMP phosphodiesterase 11A (EC 3.1.4.17)(EC 3.1.4.35)(cAMP

and cGMP phosphodiesterase 11A) [Source:UniProtKB/Swiss-Prot;Acc:P0C1Q2]

[ENSMUST00000111947]

A_55_P2166128 Gm3947 1.277 PREDICTED: Mus musculus hypothetical protein LOC100045889 (LOC100045889),

mRNA [XM_001475761]

A_55_P2077501 Gm6940 1.276 PREDICTED: Mus musculus predicted gene, EG629032 (EG629032), mRNA

[XM_901095]

A_55_P2326517 F830028O17Rik 1.275 Mus musculus 12 days embryo spinal ganglion cDNA, RIKEN full-length enriched library,

clone:D130055A15 product:unclassifiable, full insert sequence. [AK051536]

A_55_P2210213 4732423E21Rik 1.275 Mus musculus 10 days neonate skin cDNA, RIKEN full-length enriched library,

clone:4732423E21 product:unclassifiable, full insert sequence. [AK028644]

A_30_P01029774 1.273 lincRNA:chr3:51890691-51891781 reverse strand

A_55_P1979330 Dapp1 1.273 Mus musculus dual adaptor for phosphotyrosine and 3-phosphoinositides 1 (Dapp1), mRNA

[NM_011932]

A_55_P2069818 Mapk10 1.271 Mus musculus mitogen-activated protein kinase 10 (Mapk10), transcript variant 1, mRNA

[NM_009158]

A_55_P2111628 LOC676212 1.270 PREDICTED: Mus musculus similar to COLlagen family member (col-51) (LOC676212),

mRNA [XM_001474450]

A_55_P2028591 Ptprm 1.270 Mus musculus protein tyrosine phosphatase, receptor type, M (Ptprm), mRNA

[NM_008984]

A_55_P1968085 1.270

A_55_P1974627 Trappc9 1.270 Mus musculus trafficking protein particle complex 9 (Trappc9), transcript variant 5, mRNA

[NM_001164643]

A_55_P2020472 Rufy2 1.269 Mus musculus RUN and FYVE domain-containing 2 (Rufy2), mRNA [NM_027425]

A_55_P2181009 Gpr180 1.269 Mus musculus G protein-coupled receptor 180 (Gpr180), mRNA [NM_021434]

A_55_P2333434 AI956758 1.269 UI-M-GV0-chs-b-03-0-UI.r1 NIH_BMAP_GV0 Mus musculus cDNA clone

IMAGE:30542906 5', mRNA sequence [CV883746]

A_55_P1989578 1.268 Mus musculus 0 day neonate skin cDNA, RIKEN full-length enriched library,

clone:4631432E21 product:unclassifiable, full insert sequence. [AK132421]

A_66_P122927 OTTMUSG00000012483 1.268 PREDICTED: Mus musculus hypothetical LOC666709 (LOC666709),

mRNA [XM_975100]

A_55_P2062314 2900041M22Rik 1.268 Mus musculus RIKEN cDNA 2900041M22 gene (2900041M22Rik), non-coding RNA

[NR_015489]

A_30_P01024449 1.268 lincRNA:chr4:129497990-129515290 forward strand

A_52_P14666 Hnrnpa1l2 1.268 PREDICTED: Mus musculus predicted gene, EG544954, transcript variant 2 (EG544954),

mRNA [XM_888940]

A_55_P2070254 Pqbp1 1.267 Mus musculus polyglutamine binding protein 1 (Pqbp1), mRNA [NM_019478]

A_51_P440210 Prkg1 1.267 Mus musculus protein kinase, cGMP-dependent, type I (Prkg1), transcript variant 2, mRNA

[NM_011160]

A_30_P01030421 1.267 lincRNA:chr4:82156098-82196298 reverse strand

A_55_P2295951 5830427D02Rik 1.267 Mus musculus adult male thymus cDNA, RIKEN full-length enriched library,

clone:5830427D02 product:unclassifiable, full insert sequence. [AK017951]

A_55_P2052789 Cask 1.266 Mus musculus calcium/calmodulin-dependent serine protein kinase (MAGUK family)

(Cask), mRNA [NM_009806]

A_30_P01021461 1.266 lincRNA:chr2:119697845-119719245 forward strand

A_30_P01020489 1.265 lincRNA:chr3:52206874-52207385 forward strand

A_55_P1976938 4930589O11Rik 1.264 PREDICTED: Mus musculus RIKEN cDNA 4930589O11 gene (4930589O11Rik), mRNA

[XM_899432]

A_66_P136132 Gm9022 1.264 PREDICTED: Mus musculus similar to uracil DNA glycosylase (LOC675187), misc RNA

[XR_004746]

A_55_P2216079 9630055L06Rik 1.264 Mus musculus 0 day neonate eyeball cDNA, RIKEN full-length enriched library,

clone:E130006D24 product:unclassifiable, full insert sequence. [AK053276]

A_55_P2381538 A430108E01Rik 1.264 Mus musculus 13 days embryo male testis cDNA, RIKEN full-length enriched library,

clone:6030458H09 product:unclassifiable, full insert sequence. [AK031601]

A_55_P2286670 D630030B22Rik 1.263 H8189H01-5 NIA Mouse Unique Gene Set Version 2 Mus musculus cDNA clone

H8189H01 5', mRNA sequence [CK332099]

A_52_P15300 Hoxb2 1.262 Mus musculus homeobox B2 (Hoxb2), mRNA [NM_134032]

A_55_P2364703 9430037G07Rik 1.262 Mus musculus adult male liver tumor cDNA, RIKEN full-length enriched library,

clone:C730031C05 product:unclassifiable, full insert sequence. [AK050253]

A_51_P254895 Cyp4a10 1.262 Mus musculus cytochrome P450, family 4, subfamily a, polypeptide 10 (Cyp4a10), mRNA

[NM_010011]

A_55_P2185826 Cyp3a13 1.260 Mus musculus cytochrome P450, family 3, subfamily a, polypeptide 13 (Cyp3a13), mRNA

[NM_007819]

A_55_P2122255 Polm 1.259 Mus musculus mRNA for DNA polymerase mu (Polm gene). [AJ251804]

A_55_P2199202 Il22 1.259 Mus musculus interleukin 22 (Il22), mRNA [NM_016971]

A_55_P2012439 Tnfrsf19 1.259 Mus musculus tumor necrosis factor receptor superfamily, member 19 (Tnfrsf19), transcript

variant 1, mRNA [NM_013869]

A_55_P2023537 LOC100046290 1.259 PREDICTED: Mus musculus similar to ribosomal protein L21 (LOC100046290), mRNA

[XM_001476520]

A_51_P346523 Zcchc7 1.259 Mus musculus zinc finger, CCHC domain containing 7 (Zcchc7), transcript variant 2,

mRNA [NM_177027]

A_30_P01033261 1.258 lincRNA:chrX:98680220-98718445 reverse strand

A_55_P2212027 Utrn 1.258 Mus musculus utrophin (Utrn), mRNA [NM_011682]

A_52_P868555 Gm4665 1.258 PREDICTED: Mus musculus hypothetical protein LOC100043812 (LOC100043812),

mRNA [XM_001481091]

A_30_P01018862 1.258 lincRNA:chr8:13537677-13547026 reverse strand

A_55_P2364315 D030002E05Rik 1.257 Mus musculus 12 days embryo eyeball cDNA, RIKEN full-length enriched library,

clone:D230022F05 product:unclassifiable, full insert sequence. [AK051948]

A_30_P01019536 1.256 lincRNA:chr18:83530624-83534486 reverse strand

A_55_P1977766 Ttc14 1.256 Mus musculus tetratricopeptide repeat domain 14 (Ttc14), transcript variant 2, mRNA

[NM_025978]

A_30_P01028236 1.255 lincRNA:chr9:20313342-20323517 reverse strand

A_51_P294113 Chd2 1.255 Mus musculus chromodomain helicase DNA binding protein 2 (Chd2), mRNA

[NM_001081345]

A_51_P189899 Olfr1134 1.255 Mus musculus olfactory receptor 1134 (Olfr1134), mRNA [NM_147030]

A_66_P104221 Gm4122 1.255 PREDICTED: Mus musculus similar to RNP particle component (LOC100047272), mRNA

[XM_001478083]

A_51_P438805 Txnip 1.254 Mus musculus thioredoxin interacting protein (Txnip), transcript variant 1, mRNA

[NM_001009935]

A_55_P2262287 5430434G16Rik 1.254 Mus musculus 7 days neonate cerebellum cDNA, RIKEN full-length enriched library,

clone:A730047B13 product:hypothetical protein, full insert sequence. [AK139147]

A_51_P192089 2610028A01Rik 1.254 Mus musculus RIKEN cDNA 2610028A01 gene (2610028A01Rik), mRNA [NM_028228]

A_30_P01023345 1.253 lincRNA:chr4:3756167-3757483 reverse strand

A_55_P2304337 5730407M17Rik 1.252 Mus musculus 8 days embryo whole body cDNA, RIKEN full-length enriched library,

clone:5730407M17 product:unclassifiable, full insert sequence. [AK017517]

A_51_P412225 Tbc1d16 1.250 Mus musculus TBC1 domain family, member 16 (Tbc1d16), mRNA [NM_172443]

A_30_P01018763 1.250 lincRNA:chr16:59556175-59603176 reverse strand

A_55_P2103432 1.249 Putative uncharacterized proteinMCG147323 ;

[Source:UniProtKB/TrEMBL;Acc:Q8CBV7] [ENSMUST00000064248]

A_51_P163797 Bdp1 1.249 Mus musculus B double prime 1, subunit of RNA polymerase III transcription initiation

factor IIIB (Bdp1), mRNA [NM_001081061]

A_30_P01028936 1.249 lincRNA:chr17:33995525-34028025 forward strand

A_55_P1960999 Pigr 1.248 Mus musculus polymeric immunoglobulin receptor (Pigr), mRNA [NM_011082]

A_55_P2050019 Scml2 1.248 Mus musculus sex comb on midleg-like 2 (Drosophila) (Scml2), mRNA [NM_133194]

A_55_P2041457 Gm3195 1.248 PREDICTED: Mus musculus hypothetical protein LOC100041198 (LOC100041198),

mRNA [XM_001475948]

A_55_P2251181 2900076A13Rik 1.248 Mus musculus adult male hippocampus cDNA, RIKEN full-length enriched library,

clone:2900076A13 product:unclassifiable, full insert sequence. [AK013795]

A_55_P2260052 LOC654469 1.247 Mus musculus adult male stomach cDNA, RIKEN full-length enriched library,

clone:2210413J24 product:unclassifiable, full insert sequence. [AK131895]

A_55_P2072940 1.247 hypothetical protein LOC231668 [Source:RefSeq peptide;Acc:NP_001028483]

[ENSMUST00000100833]

A_30_P01030539 1.246 lincRNA:chr1:85300475-85331475 reverse strand

A_55_P2245422 D11Wsu173e 1.246 Mus musculus 0 day neonate thymus cDNA, RIKEN full-length enriched library,

clone:A430087M13 product:unclassifiable, full insert sequence. [AK138827]

A_30_P01025309 1.246 lincRNA:chr5:137392195-137426421 forward strand

A_55_P1967095 LOC100044816 1.246 PREDICTED: Mus musculus similar to cyclic nucleotide gated channel beta 1

(LOC100044816), mRNA [XM_001473112]

A_51_P398191 Auts2 1.246 Mus musculus autism susceptibility candidate 2 (Auts2), mRNA [NM_177047]

A_51_P471791 St8sia6 1.244 Mus musculus ST8 alpha-N-acetyl-neuraminide alpha-2,8-sialyltransferase 6 (St8sia6),

mRNA [NM_145838]

A_55_P2128220 Gm7496 1.244 PREDICTED: Mus musculus predicted gene, EG665109 (EG665109), mRNA

[XM_975494]

A_55_P1991239 1.243 Putative uncharacterized protein Fragment [Source:UniProtKB/TrEMBL;Acc:Q8BQ66]

[ENSMUST00000061906]

A_30_P01025841 1.243 lincRNA:chr13:62376235-62376890 forward strand

A_30_P01018966 1.243 lincRNA:chr13:70745306-70837331 reverse strand

A_55_P2340950 AY512938 1.243 Mus musculus clone NIA:H4075D09 unknown mRNA. [AY512938]

A_66_P140533 Gm3813 1.243 PREDICTED: Mus musculus similar to Pro-Pol-dUTPase polyprotein; RNaseH; dUTPase;

integrase; protease; reverse transcriptase (LOC100042383), mRNA [XM_001478088]

A_55_P2011567 LOC634184 1.242 PREDICTED: Mus musculus similar to T-cell receptor alpha precursor (LOC634184),

mRNA [XM_908838]

A_55_P2227154 A530041M06Rik 1.241 Mus musculus adult male aorta and vein cDNA, RIKEN full-length enriched library,

clone:A530041M06 product:unclassifiable, full insert sequence. [AK040901]

A_51_P155465 Cuzd1 1.241 Mus musculus CUB and zona pellucida-like domains 1 (Cuzd1), mRNA [NM_008411]

A_66_P112371 Cacna1i 1.240 Mus musculus calcium channel, voltage-dependent, alpha 1I subunit (Cacna1i), mRNA

[NM_001044308]

A_30_P01032760 1.239 lincRNA:chr4:126710640-126721240 forward strand

A_55_P2049279 Rnf150 1.239 Mus musculus ring finger protein 150 (Rnf150), mRNA [NM_177378]

A_55_P1968883 LOC100045940 1.239 PREDICTED: Mus musculus similar to transcription factor mammalian MafA

(LOC100045940), mRNA [XM_001475220]

A_55_P1989865 Thrap3 1.239 Mus musculus thyroid hormone receptor associated protein 3 (Thrap3), mRNA

[NM_146153]

A_52_P543079 Dhtkd1 1.238 Mus musculus dehydrogenase E1 and transketolase domain containing 1 (Dhtkd1), mRNA

[NM_001081131]

A_51_P355301 Cyp3a11 1.238 Mus musculus cytochrome P450, family 3, subfamily a, polypeptide 11 (Cyp3a11), mRNA

[NM_007818]

A_30_P01027323 1.238 lincRNA:chr18:36449530-36456800 reverse strand

A_55_P2134712 Gm3781 1.237 PREDICTED: Mus musculus similar to pORF2 (LOC100042306), mRNA

[XM_001478003]

A_55_P2021780 Gm9897 1.237 Mus musculus predicted gene 9897 (Gm9897), nuclear gene encoding mitochondrial

protein, mRNA [NM_001042670]

A_30_P01029729 1.237 lincRNA:chr11:58136882-58143829 reverse strand

A_30_P01020219 1.236 lincRNA:chr15:43363046-43364763 forward strand

A_55_P2017845 Ehbp1 1.236 Mus musculus EH domain binding protein 1 (Ehbp1), mRNA [NM_153078]

A_52_P634122 Rexo1 1.235 Mus musculus REX1, RNA exonuclease 1 homolog (S. cerevisiae) (Rexo1), transcript

variant 1, mRNA [NM_025852]

A_66_P113505 Gm3006 1.234 PREDICTED: Mus musculus similar to pORF2 (LOC100040860), mRNA

[XM_001475977]

A_30_P01032449 1.233 lincRNA:chr8:72105642-72105922 reverse strand

A_55_P2069052 1.231 Sacsin [Source:UniProtKB/Swiss-Prot;Acc:Q9JLC8] [ENSMUST00000119509]

A_30_P01026332 1.231 lincRNA:chr4:126710640-126721240 forward strand

A_66_P136095 Ctnna3 1.230 Mus musculus catenin (cadherin associated protein), alpha 3 (Ctnna3), transcript variant 1,

mRNA [NM_001164376]

A_55_P2376363 4930432F04Rik 1.230 Mus musculus RIKEN cDNA 4930432F04 gene, mRNA (cDNA clone MGC:28765

IMAGE:4485816), complete cds. [BC016220]

A_30_P01031167 1.230 lincRNA:chr9:58197113-58200614 forward strand

A_55_P2167922 Dmd 1.229 Mus musculus dystrophin, muscular dystrophy (Dmd), mRNA [NM_007868]

A_55_P2222437 D430040D24Rik 1.229 Mus musculus 13 days embryo lung cDNA, RIKEN full-length enriched library,

clone:D430047J20 product:unclassifiable, full insert sequence. [AK052542]

A_55_P2065334 Gm14430 1.229 Mus musculus predicted gene 14430 (Gm14430), mRNA [NM_001100415]

A_66_P123735 1.229 Mus musculus 12 days embryo whole body cDNA, RIKEN full-length enriched library,

clone:E970027J19 product:hypothetical L1 transposable element containing protein, full

insert sequence. [AK143547]

A_55_P2180736 Zfp740 1.229 Mus musculus zinc finger protein 740 (Zfp740), mRNA [NM_153194]

A_51_P436727 Arrb1 1.228 Mus musculus arrestin, beta 1 (Arrb1), transcript variant a, mRNA [NM_177231]

A_30_P01024460 1.228 lincRNA:chr8:59966759-59989065 reverse strand

A_55_P2270740 8030462D06Rik 1.227 Mus musculus 15 days embryo male testis cDNA, RIKEN full-length enriched library,

clone:8030462D06 product:unclassifiable, full insert sequence. [AK020212]

A_55_P2009841 Pld5 1.227 Mus musculus phospholipase D family, member 5 (Pld5), mRNA [NM_176916]

A_30_P01024283 1.225 lincRNA:chr5:13067925-13096000 reverse strand

A_51_P433000 LOC100044854 1.225 PREDICTED: Mus musculus similar to chromatin modifying protein 4C (LOC100044854),

mRNA [XM_001473048]

A_30_P01019979 1.223 lincRNA:chr19:54011403-54018978 reverse strand

A_55_P1959379 BC080695 1.222 Mus musculus cDNA sequence BC080695 (BC080695), mRNA [NM_001007579]

A_55_P1998721 1.222 Putative uncharacterized protein Fragment [Source:UniProtKB/TrEMBL;Acc:Q3UUZ7]

[ENSMUST00000099502]

A_30_P01025117 1.222 lincRNA:chr9:88341846-88342499 reverse strand

A_30_P01022496 1.221 lincRNA:chr17:26911032-26912366 forward strand

A_55_P2295786 B230380D07Rik 1.221 Mus musculus RIKEN cDNA B230380D07 gene (B230380D07Rik), mRNA [NM_172772]

A_55_P1984846 LOC100048079 1.220 PREDICTED: Mus musculus similar to Zinc finger and BTB domain containing 1

(LOC100048079), mRNA [XM_001479478]

A_30_P01032288 1.220 lincRNA:chr17:31694070-31696057 forward strand

A_30_P01018499 1.220 lincRNA:chr18:61809278-61825191 reverse strand

A_55_P2328920 Tle4 1.220 Mus musculus transducin-like enhancer of split 4, homolog of Drosophila E(spl) (Tle4),

mRNA [NM_011600]

A_30_P01030969 1.220 lincRNA:chr1:175660642-175671878 reverse strand

A_55_P2182222 Rab3c 1.219 Mus musculus RAB3C, member RAS oncogene family (Rab3c), mRNA [NM_023852]

A_30_P01018463 1.218 lincRNA:chr4:59264275-59287850 reverse strand

A_55_P1997951 Htatsf1 1.218 Mus musculus HIV TAT specific factor 1 (Htatsf1), mRNA [NM_028242]

A_65_P19933 Zdhhc23 1.218 Probable palmitoyltransferase ZDHHC23 (EC 2.3.1.-)(Zinc finger DHHC

domain-containing protein 23)(DHHC-23)(DHHC-containing protein 11)

[Source:UniProtKB/Swiss-Prot;Acc:Q5Y5T3] [ENSMUST00000036321]

A_30_P01024376 1.217 lincRNA:chr1:134237581-134248506 reverse strand

A_55_P2042675 Ttc28 1.217 Mus musculus tetratricopeptide repeat domain 28 (Ttc28), mRNA [NM_024477]

A_55_P2030334 D0Kist3 1.217 Mus musculus CAG trinucleotide repeat mRNA, partial sequence. [U80890]

A_52_P279184 Srp54a 1.216 Mus musculus signal recognition particle 54A (Srp54a), mRNA [NM_011899]

A_55_P2095854 Rab3c 1.216 Mus musculus RAB3C, member RAS oncogene family (Rab3c), mRNA [NM_023852]

A_52_P207982 Gtf3c2 1.215 Mus musculus general transcription factor IIIC, polypeptide 2, beta (Gtf3c2), mRNA

[NM_027901]

A_55_P1962219 Prima1 1.215 Mus musculus proline rich membrane anchor 1 (Prima1), mRNA [NM_133364]

A_55_P2411907 B230213L16Rik 1.215 Mus musculus visual cortex cDNA, RIKEN full-length enriched library,

clone:K530030A20 product:similar to Hypothetical phenylalanine-rich region containing

protein [Mus musculus], full insert sequence. [AK159005]

A_65_P13270 Csnk1g1 1.214 Mus musculus casein kinase 1, gamma 1 (Csnk1g1), mRNA [NM_173185]

A_55_P2126627 Phf20l1 1.214 Mus musculus PHD finger protein 20-like 1 (Phf20l1), mRNA [NM_001081409]

A_30_P01020809 1.214 lincRNA:chr2:155269145-155303251 reverse strand

A_55_P2059105 1.213 Putative uncharacterized protein [Source:UniProtKB/TrEMBL;Acc:Q8C4M2]

[ENSMUST00000110230]

A_30_P01028690 1.213 lincRNA:chr4:126009815-126015875 forward strand

A_55_P2258612 LOC433461 1.213 Mus musculus 12 days embryo eyeball cDNA, RIKEN full-length enriched library,

clone:D230021J12 product:unclassifiable, full insert sequence. [AK051942]

A_55_P1979699 Spop 1.213 Mus musculus speckle-type POZ protein (Spop), mRNA [NM_025287]

A_30_P01024680 1.212 lincRNA:chr1:137482591-137625604 forward strand

A_55_P2113165 Camkk1 1.212 Mus musculus calcium/calmodulin-dependent protein kinase kinase 1, alpha (Camkk1),

mRNA [NM_018883]

A_51_P210143 Syne2 1.211 Mus musculus synaptic nuclear envelope 2 (Syne2), mRNA [NM_001005510]

A_30_P01018212 1.211 lincRNA:chr3:37543528-37546874 forward strand

A_55_P2339379 Sass6 1.210 Mus musculus spindle assembly 6 homolog (C. elegans) (Sass6), mRNA [NM_028349]

A_55_P2149288 Cmya5 1.210 Mus musculus cardiomyopathy associated 5 (Cmya5), mRNA [NM_023821]

A_55_P2335718 Wdr45l 1.209 Mus musculus 15 days embryo head cDNA, RIKEN full-length enriched library,

clone:D930015F23 product:RIKEN cDNA 0610008N23 gene, full insert sequence.

[AK086235]

A_55_P2121484 Smo 1.209 Mus musculus smoothened homolog (Drosophila) (Smo), mRNA [NM_176996]

A_30_P01018718 1.209 lincRNA:chr14:65431652-65437902 reverse strand

A_66_P115443 Snx4 1.208 Mus musculus sorting nexin 4 (Snx4), mRNA [NM_080557]

A_55_P1962621 1.208 chitinase like [Source:RefSeq peptide;Acc:NP_001074285] [ENSMUST00000054973]

A_55_P2119877 1.208 Splicing factor, arginine/serine-rich 18 (Splicing factor, arginine/serine-rich

130)(Serine-arginine-rich-splicing regulatory protein 130)(SRrp130)

[Source:UniProtKB/Swiss-Prot;Acc:A2AJT4] [ENSMUST00000108229]

A_55_P2102759 Gm2459 1.207 PREDICTED: Mus musculus similar to Hmgb1 protein (LOC100039851), mRNA

[XM_001473665]

A_55_P2016252 Ctnnal1 1.206 Mus musculus catenin (cadherin associated protein), alpha-like 1 (Ctnnal1), mRNA

[NM_018761]

A_30_P01030325 1.206 lincRNA:chr2:155269145-155303251 reverse strand

A_52_P52263 D17Wsu92e 1.206 Mus musculus DNA segment, Chr 17, Wayne State University 92, expressed (D17Wsu92e),

transcript variant 2, mRNA [NM_001044719]

A_55_P2135220 Gm2204 1.206 PREDICTED: Mus musculus similar to retinitis pigmentosa GTPase regulator interacting

protein 1 (LOC100039391), mRNA [XM_001472809]

A_52_P159256 Lpp 1.206 Mus musculus LIM domain containing preferred translocation partner in lipoma (Lpp),

transcript variant 1, mRNA [NM_178665]

A_52_P188425 Prosc 1.205 Mus musculus proline synthetase co-transcribed (Prosc), transcript variant 2, mRNA

[NM_001039077]

A_30_P01030510 1.205 lincRNA:chr19:57426515-57435165 reverse strand

A_55_P1955528 1.205

A_55_P2146384 Filip1 1.204 Mus musculus filamin A interacting protein 1 (Filip1), mRNA [NM_001081243]

A_55_P2001218 Cd80 1.204 Mus musculus CD80 antigen (Cd80), mRNA [NM_009855]

A_51_P311904 Ptk2b 1.203 Mus musculus PTK2 protein tyrosine kinase 2 beta (Ptk2b), transcript variant 3, mRNA

[NM_172498]

A_55_P2082314 LOC100046040 1.203 PREDICTED: Mus musculus hypothetical protein LOC100046040 (LOC100046040),

mRNA [XM_001476290]

A_30_P01031333 1.202 lincRNA:chr13:12792150-12869825 forward strand

A_55_P2066453 1.202 Putative uncharacterized proteinMCG147252 ;

[Source:UniProtKB/TrEMBL;Acc:Q8C4K5] [ENSMUST00000076071]

A_55_P2020054 2610035D17Rik 1.201 Mus musculus 13 days embryo lung cDNA, RIKEN full-length enriched library,

clone:D430003N19 product:hypothetical protein, full insert sequence. [AK164572]

A_30_P01033567 1.201 lincRNA:chr19:5771425-5848475 forward strand

A_55_P2154740 LOC100046007 1.201 PREDICTED: Mus musculus similar to cyclic nucleotide gated channel beta 1

(LOC100046007), mRNA [XM_001475346]

A_51_P276715 Olfr1350 1.201 Mus musculus olfactory receptor 1350 (Olfr1350), mRNA [NM_146389]

A_51_P136521 Lypd2 1.201 Mus musculus Ly6/Plaur domain containing 2 (Lypd2), mRNA [NM_026671]
